# Self-Assembly of Binderless MXene Aerogel for Multiple-Scenario and Responsive Phase Change Composites with Ultrahigh Thermal Energy Storage Density and Exceptional Electromagnetic Interference Shielding

**DOI:** 10.1007/s40820-023-01288-y

**Published:** 2023-12-18

**Authors:** Chuanbiao Zhu, Yurong Hao, Hao Wu, Mengni Chen, Bingqing Quan, Shuang Liu, Xinpeng Hu, Shilong Liu, Qinghong Ji, Xiang Lu, Jinping Qu

**Affiliations:** 1 Key Laboratory of Material Chemistry for Energy Conversion and Storage of Ministry of Education, School of Chemistry and Chemical Engineering, Huazhong University of Science and Technology, Wuhan, 430074 People’s Republic of China; 2 Qingdao Mental Health Center, Qingdao, 266034 People’s Republic of China; 3 Hubei Engineering Research Center for Biomaterials and Medical Protective Materials, Huazhong University of Science and Technology, Wuhan, 430074 People’s Republic of China; 4 Hubei Key Laboratory of Material Chemistry and Service Failure, School of Chemistry and Chemical Engineering, Huazhong University of Science and Technology, Wuhan, 430074 People’s Republic of China; 5 National Engineering Research Center of Novel Equipment for Polymer Processing, Key Laboratory of Polymer Processing Engineering (South China University of Technology), Ministry of Education, Guangdong Provincial Key Laboratory of Technique and Equipment for Macromolecular Advanced Manufacturing, School of Mechanical and Automotive Engineering, South China University of Technology, Guangzhou, 510641 People’s Republic of China

**Keywords:** Self-assembly, Multiple-scenario, Phase change composites, Thermal energy storage, Electromagnetic interference shielding

## Abstract

The severe dependence of traditional phase change materials (PCMs) on the temperature-response and lattice deficiencies in versatility cannot satisfy demand for using such materials in complex application scenarios. Here, we introduced metal ions to induce the self-assembly of MXene nanosheets and achieve their ordered arrangement by combining suction filtration and rapid freezing. Subsequently, a series of MXene/ K^+^/paraffin wax (PW) phase change composites (PCCs) were obtained via vacuum impregnation in molten PW. The prepared MXene-based PCCs showed versatile applications from macroscale technologies, successfully transforming solar, electric, and magnetic energy into thermal energy stored as latent heat in the PCCs. Moreover, due to the absence of binder in the MXene-based aerogel, MK3@PW exhibits a prime solar–thermal conversion efficiency (98.4%). Notably, MK3@PW can further convert the collected heat energy into electric energy through thermoelectric equipment and realize favorable solar–thermal–electric conversion (producing 206 mV of voltage with light radiation intensity of 200 mw cm^−2^). An excellent Joule heat performance (reaching 105 °C with an input voltage of 2.5 V) and responsive magnetic–thermal conversion behavior (a charging time of 11.8 s can achieve a thermal insulation effect of 285 s) for contactless thermotherapy were also demonstrated by the MK3@PW. Specifically, as a result of the ordered arrangement of MXene nanosheet self-assembly induced by potassium ions, MK3@PW PCC exhibits a higher electromagnetic shielding efficiency value (57.7 dB) than pure MXene aerogel/PW PCC (29.8 dB) with the same MXene mass. This work presents an opportunity for the multi-scene response and practical application of PCMs that satisfy demand of next-generation multifunctional PCCs.

**Supplementary Information:**

The online version contains supplementary material available at 10.1007/s40820-023-01288-y.

## Highlights

- This work proposes a tactic for improving the efficiency of thermal energy conversion and expanding the application scenarios of phase change materials by constructing non-binder and oriented MXene-K^+^ aerogel.
- The prepared phase change composites (PCCs) can rapidly transform solar, electric, magnetic energy into latent heat for keeping warm, power generation, and thermal physiotherapy.
- Owing to the suggested tactic, the prepared PCCs achieves ultrahigh energy storage density and realize 99.9998% electromagnetic wave energy attenuation.

**Supplementary Information:**

The online version contains supplementary material available at 10.1007/s40820-023-01288-y.

## Graphical abstract

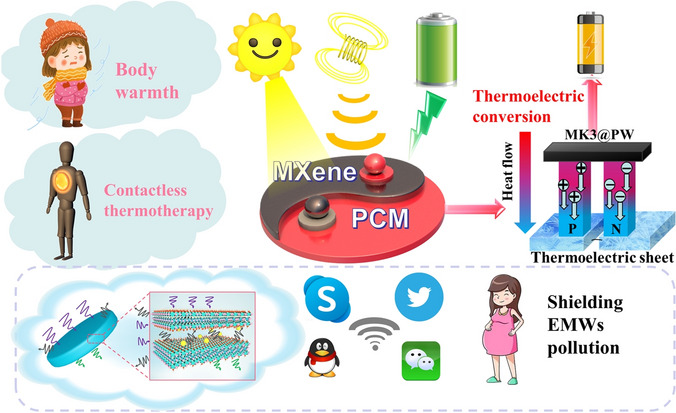

## Introduction

With the increasing human demand for the comfortable thermal environment, it is really considerable to maintain a relatively constant, comfortable body temperature to enable various bodily functions [1, 2], while most of the fossil fuel is consumed for the thermal management of human daily life [3], accounting for 40% of the world’s total energy consumption energy [4]. In the current complex application environment, developing an energy storage technology which can achieve efficient thermal energy conversion and utilization has become a challenge to be addressed [5]. On the other hand, with the popularization of miniature precision electronic equipment, such as 5G smartphones and computers, generating a large number of electromagnetic waves (EMWs), health issues caused by EMWs have become increasingly urgent [6]. Consequently, multifunctional portable device which can energy-savingly and timely provide thermal management for human body temperature and protect human body from EMW pollution are necessary for meeting energy conservation and high-quality life demanding.

Latent heat storage (LHS) technology [7, 8], based on phase change materials (PCMs), is considered a promising and cost-effective energy medium for thermal management because it can reversibly absorb and release heat with minimal temperature variation during the phase change process [9, 10]. Currently, among the more than 6,000 PCMs that have been researched, paraffin wax (PW) is deemed an ideal substitute for human thermal management materials due to its low cost, high phase change enthalpy, moderate phase change temperature, etc. [11–13]. Nevertheless, as a classic solid–liquid organic PCM, its tendency to leak is a long-lasting choke point in industry utilization scenarios. Moreover, energy sources such as solar energy, electric energy, and magnetic energy can be converted into thermal energy, but traditional organic PCMs are only able to respond to variations in temperature and directly store the heat, which extremely limits their application scenario in saving heat from more sources except for just temperature. To overcome these challenges, incorporating PCMs into aerogels to prepare shape-stabilized phase change composites (PCCs) is a feasible solution, aerogel-based PCCs is considered a leading-edge concept, to endow them with the abundant functions of PCCs [14, 15]. On the one hand, the aerogel is accepted as an ideal class of supporting material that can effectively prevent PCMs from leakage due to strong capillary forces and surface tension. Due to their ultrahigh porosity and specific surface area, PCMs can achieve extreme adsorption in aerogels, thereby obtaining ultrahigh energy storage density. On the other hand, the functions of multifunctional aerogel materials provide possibilities for improving thermal energy storage efficiency, converting multiple energy sources to thermal energy, shielding EMW pollution, and broadening the application scenario of PCCs.

MXene, a type of metal carbide or nitride material with a 2D layer structure, compared with previously reported supporting materials for PCMs (including copper nanowire aerogel [16], silver nanowire aerogel [17], cellulose aerogel [18], carbon nanotube aerogel [19], graphene aerogel [20], silica aerogel [21], and boron nitride aerogel [22, 23]), is considered a better candidate for supporting PCMs due to its synergistic functions of numerous oxygen-containing groups [24], superior electrical conductivities (10,000 S cm^−1^) [25, 26], high surface area [27, 28], and nearly 100% internal solar–thermal conversion efficiency [29, 30]. What’s more, MXenes also exhibit extremely outstanding electromagnetic shielding efficiency, and it has been suggested that the combination of MXene aerogels and PCMs may further protect human bodies and electronic devices from electromagnetic pollution except thermal runaway. To further advance for multifunctional PCCs, several works have infiltrated PCMs into MXene/polyvinyl alcohol [31] (PVA) scaffolds and MXene/cellulose nanofibrous (CNFs) aerogels [32], which both attained higher thermal storage efficiency and conversion abilities between thermal energy and other energies. Specifically, the MXene/PVA skeleton provides the PCCs with properties that can be driven by solar energy and effectively shield electromagnetic waves. The above fabrication methods have successfully produced MXene-based PCCs. However, their loading rate of PCMs, energy storage efficiency, and EMI shielding efficiency are generally sacrificed due to external binders and additional functionalization [33–35]. Directly freeze-drying pure MXene suspensions can obtain binder-free MXene aerogels, but these fabricated MXene aerogels normally have low electrical conductivity and poor structural stability due to weak interlayer interactions. Although foaming an MXene film at high temperature using gas generated from hydrazine hydrates can also obtain an MXene aerogel [36], this procedure requires highly toxic hydrazine hydrates, and the high temperature treatment of the procedure will inevitably expose MXene to the risk of oxidation and lead to the waste of its original excellent performance. Therefore, to maximize the energy storage efficiency, conversion efficiency of other energy to thermal energy and electromagnetic interference efficiency of shape-stabilized PCCs, it is necessary to build a promotable method without incorporating binders or spacers to construct MXene-based aerogels for rapid latent heat energy storage efficiency and stringent energy efficiency requirements on next-generation multifunctional PCCs.

**Scheme 1:**
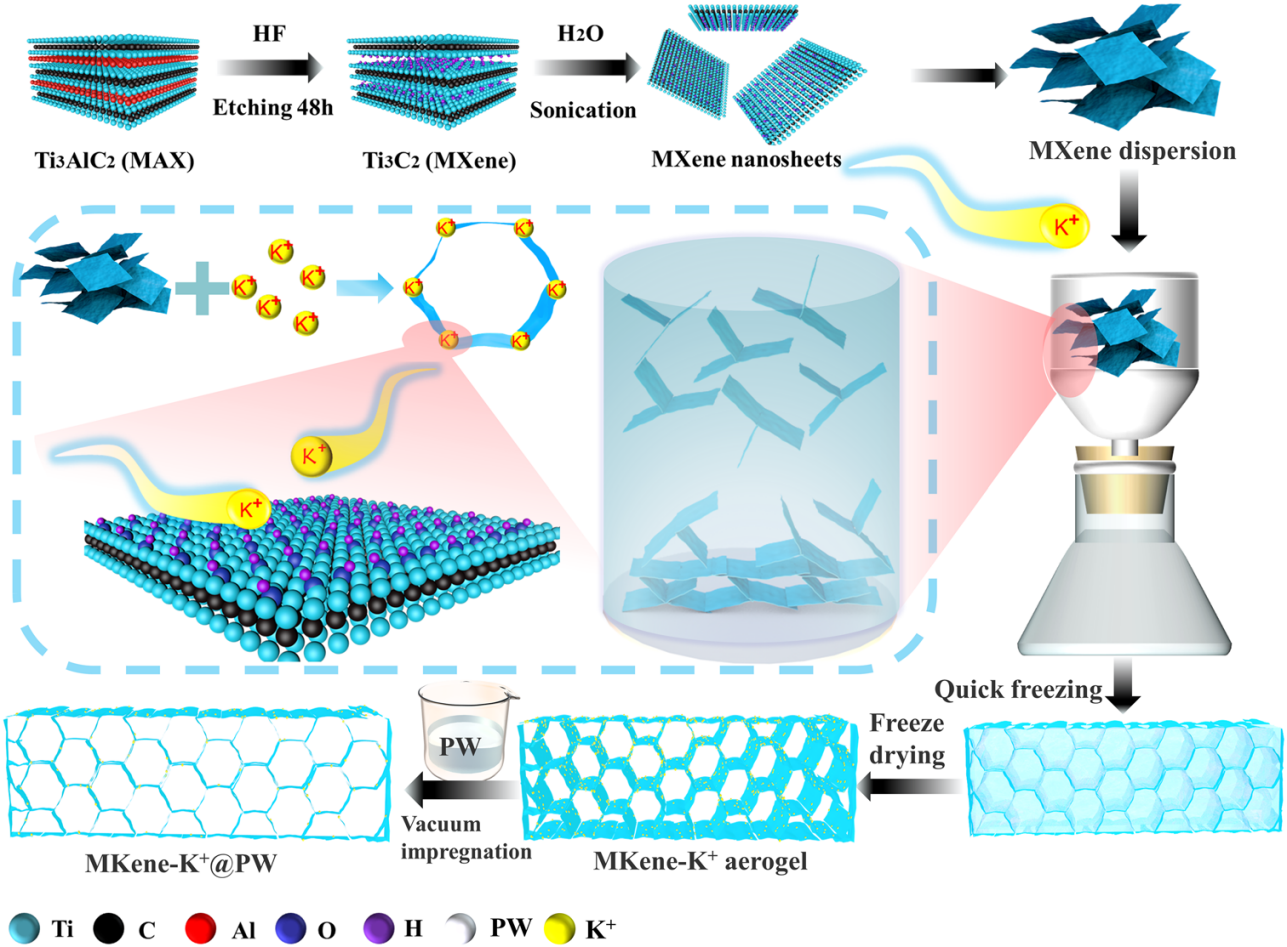
The preparation process of MXene-K^+^@PW PCCs

Based on these considerations, we introduced metal ions to induce self-assembly of MXene nanosheets and achieve ordered an arrangement of MXene nanosheets with the combination of suction filtration and rapid freezing (Scheme 1). Benefiting from the strong binding energy between metal ions and –OH groups on the MXene nanosheets, the metal ions acted as binding sites enhancing the interconnections of MXene nanosheets and inducing the formation of a stable conductive network. Subsequently, a series of MXene-K^+^@PW PCCs were obtained via vacuum impregnation in molten PW. On the one hand, the high porosity of MXene-K^+^ aerogel realizes the ultrahigh mass loading of PW in PCCs, as a consequence, MXene-K^+^@PW achieves an extremely high energy density. On the other hand, MXene-K^+^@PW PCCs demonstrates the maximum conductively and light absorption as generated by the MXene because of its absence of binders. Therefore, the MXene-K^+^@PW PCCs exhibit high light-to-thermal conversion efficiency, notably, MXene-K^+^@PW can further convert the collected heat energy into electric energy through thermoelectric equipment and realize good solar–thermal–electric conversion. Excellent Joule heat performance and responsive magnetic–thermal conversion behavior for contactless thermotherapy were also exhibited by MXene-K^+^@PW. In addition, as a result of the ordered arrangement of MXene nanosheets, MXene-K^+^@PW PCCs exhibit exceptional electromagnetic shielding efficiency values, which can inhibit 99.9998% of the incident EMW with only 0.0002% transmission. Therefore, these multi-scene responsive portable devices provide both a stable heat source and EMI shielding to meet the demand for next-generation multifunctional PCCs.

## Experimental Section

### Preparation of the MXene Suspension

Ti_3_X_2_ MXene suspension was prepared according to our previous report [37].

### Preparation of the MXene-K^+^ Aerogel

First, KCl solution (10 mL) was added to 10 mL MXene suspension (10 mg mL^−1^). Then, the MXene microgel dispersion was vacuum filtered through a nylon filtration membrane (0.45 μm pore size). The vacuum was disconnected once there was no obvious residual dispersion on the filter membrane. Next, the MXene microgel was directly immersed in liquid nitrogen. Finally, the MXene-K^+^ aerogel was obtained by freeze-drying immediately after the freezing in liquid nitrogen. According to the mass ratios of KCl to MXene of 1:1, 2:1, 4:1, and 8:1, the different degrees of MXene aerogels were named MK1, MK2, MK3, and MK4, respectively.

### Preparation of MXene-K^+^@PW PCCs

The MXene-K^+^@PW PCCs were prepared by vacuum impregnation measure. Then, MXene-K^+^ aerogels were immersed into molten PW for vacuum impregnation (the vacuum degree was − 0.1 MPa) at 80 °C for 10 h. After, these composites were put on filter papers in an oven (80 °C) to expunge the redundant PW on the surfaces of the samples. Correspondingly, the PCCs obtained from MK1, MK2, MK3, and MK4 were named MK1@PW, MK2@PW, MK3@PW, and MK4@PW, respectively.

## Results and Discussion

### Morphology and Formation Mechanism of the MXene-K^+^ Aerogel

**Fig. 1:**
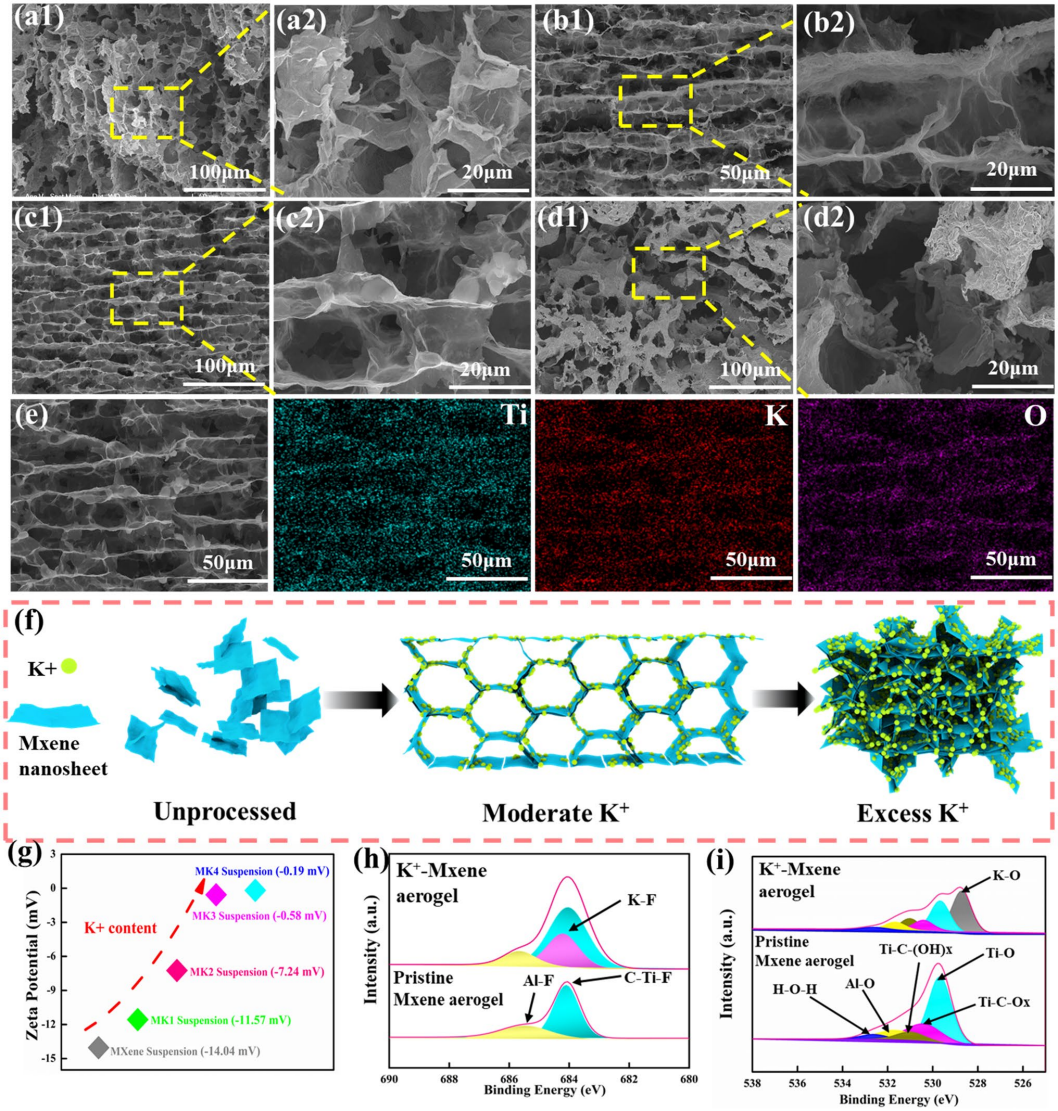
Cross-sectional SEM images of MK1 aerogel (**a1**, **a2**), MK2 aerogel (**b1**, **b2**), MK3 aerogel (**c1**, **c2**), and MK4 aerogel (**d1**, **d2**); **e** SEM images of MK3 aerogel and EDS mappings of Ti, K, and O elements. **f** Assembly structure with different K^+^ content. **g** Zeta potential of MXene suspension and different K^+^ content MXene-K^+^ suspension. **h** F 1*s* spectra of MXene aerogel and MK3 aerogel. **i** O 1*s* spectra of MXene aerogel and MK3 aerogel

Scanning electron microscopy (SEM) was used to observe the structure of the MXene-K^+^ aerogel first. As a control, the MXene aerogel without K^+^ treatment is formed a porous structure with poor connectivity (Fig. S1). Instead, all MXene-K^+^ aerogels displayed an interlinked 3D network (Fig. 1a–d), and the proportion of the weight of KCl to MXene is a crucial factor in the MXene nanosheets arrangement. As the weight proportion of MXene to KCl increased from 1:1 to 1:8, the aggregation of the MXene nanosheets gradually formed along with the formation of thick and tightly stacked skeletons, which was also accompanied by an increase in the density of the MXene-K^+^ aerogel from 8.65 to 80.79 mg cm^−3^ (Fig. S2). Moreover, the channels of MXene-K^+^ aerogels changed from no orientation to horizontal alignment and then tended to disorder. At a lower K^+^ proportion, K^+^ is not enough to cross-link MXene nanosheets to form stable micro gel (Fig. S3), and MK1 aerogel displays a typical honeycomb structure (Fig. 1a), MXene nanosheets disorderly stacked and bridge to each other. When the proportion continued to increase to 1:2 and 1:4, the cross sections of MK2 (Fig. 1b) and MK3 (Fig. 1c) aerogel presented a long-range regular horizontally aligned MXene nanosheets orientation layered porous structure. Their layer spacing size is about 10 μm, and there is an appropriate amount of MXene nanosheets lapped between layered frameworks like bridges. Moreover, according to the energy-dispersive X-ray spectroscopy (EDX) mapping of the cross section of MK3 aerogel (Fig. 1e), K^+^ exhibited a uniform distribution. With further increase of KCl, the MXene nanosheets orientation disappeared when the proportion of weight of MXene to KCl reaches 1:8, and the serious agglomeration of MXene nanosheets was observed in the MK4 aerogel (Fig. 1d).

It can be inferred that the arrangement of MXene nanosheets is generated by the comprehensive effects of suction pressure and K^+^ adsorption. The K^+^-induced gelation of MXene nanosheets can be divided into two steps, which is similar to the GO gelation process [38]. First, the combination between K^+^ and the groups on the surface of MXene disrupted the electrostatic balance among the MXene suspensions, as the MXene nanosheets are negatively charged (-14.04 mV), and the K^+^ ions are positively charged. Then, the K^+^ ions act as binding sites to induce MXene nanosheet self-assembly. As intended, the potential of the MXene-K^+^ suspension is gradually less negative than that of the original MXene suspension (Fig. 1f), and MXene surface charge is completely neutralized (-0.19 mV) after the mass ratio of MXene to KCl reaches 1:8, which demonstrates the successful flocculation and the aggregation of the MXene nanosheets. Figure 1g illustrates the growth process of MXene nanosheets to microgels. Without or with less K^+^ added into the MXene suspension, MXene nanosheets are randomly and stably distributed in suspension. Then, as KCl content increased, the particle size increased, and the 2D lamellar sheets changed to 3D micro networks. After attachment of the moderate mass ratio of K^+^, MXene nanosheets self-assemble into layered porous structures with a horizontal orientation, while excessive K^+^ accumulation will rapidly destroy the electrostatic balance on the MXene surface, leading to severe aggregation behavior. More importantly, in the F 1*s* (Fig. 1h) and O 1*s* spectra (Fig. 1i), the characteristic peaks representing K–F bonds and K–O bonds were observed at 628.6 and 528.9 eV, respectively, demonstrating crosslinking induced by K^+^ with –OH groups and –F groups of MXene nanosheets that resulted in the high structural stability of MXene-K^+^ aerogels [39].

### Morphology and Packaging Properties of MXene-KCl@PW PCCs

**Fig. 2:**
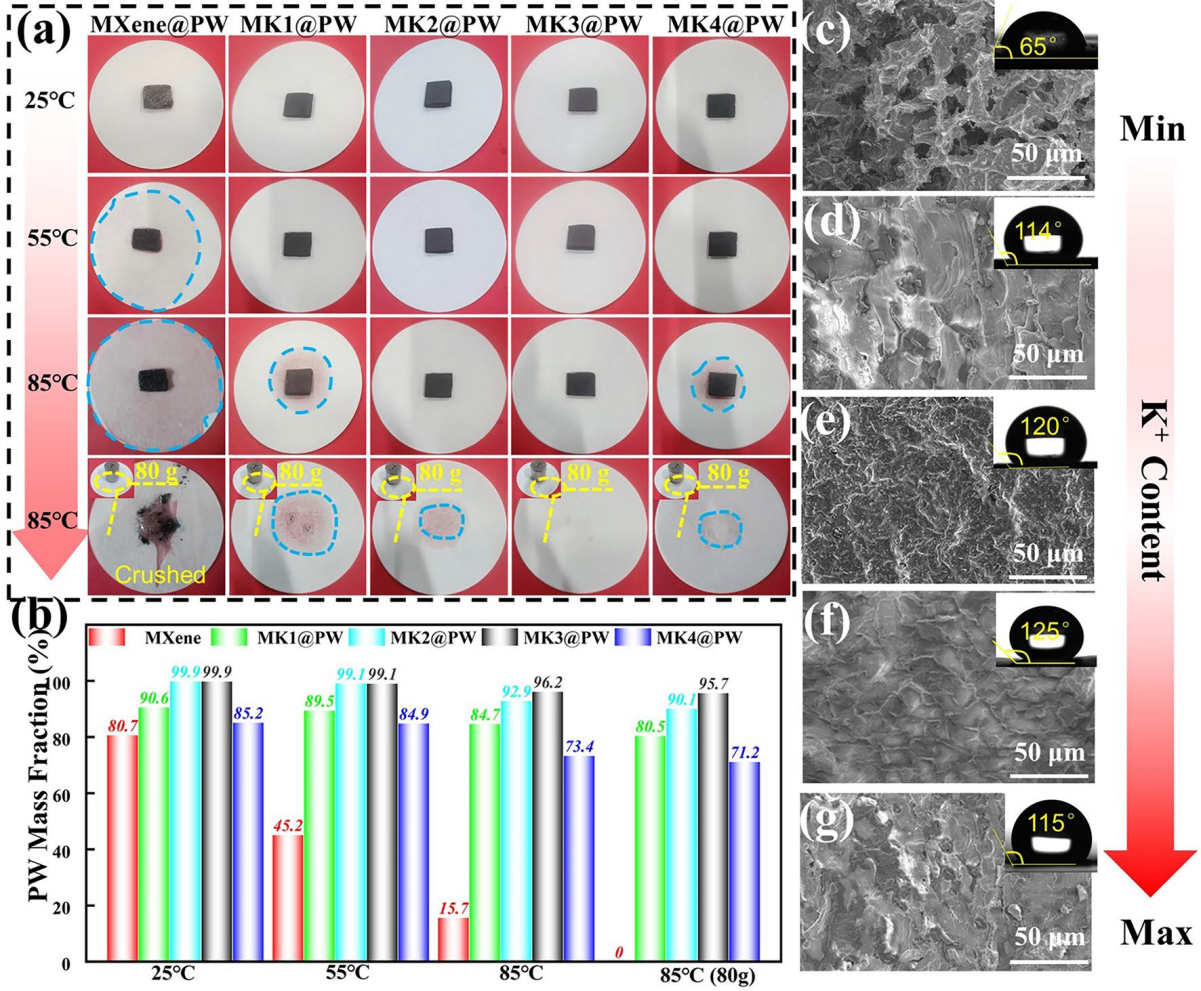
**a** Leakage test of MXene@PW, MK1@PW, MK2@PW, MK3@PW, MK4@PW under different temperature and pressure; **b** PW mass loading after different temperature and pressure leakage test; upper surface SEM images and water contact angle of **c** MXene@PW, **d** MK1@PW, **e** MK2@PW, **f** MK3@PW, **g** MK4@PW after leakage test under 85 °C for 10 h

Shape stability is an important factor in measuring the service stability of PCCs. To explore the shape stability of PW in the MXene-K^+^@PW PCCs at high temperatures and external pressure, samples were placed on filter paper in an oven. The leakage behavior at different temperature and pressure was recorded by a camera. As shown in Fig. 2a, in a leakage test at 55 °C, MXene@PW PCCs showed a poor shape stabilization ability for there are apparent leakage traces, its PW mass loading decreased from 80.7 to 45.2 wt%. In sharp contrast, few leakage traces can be observed underneath every MXene-K^+^@PW PCC after 4 h at 55 °C. Then, with the temperature attached at 85 °C, MXene@PW PCC displayed more serious leakage and its PW mass loading ratio rapidly dropped to 15.7 wt%. Simultaneously, MK1@PW and MK4@PW showed a slight leakage phenomenon, suggesting that insufficient K^+^ and excessive K^+^ will lead to incomplete crosslinking and severe agglomeration, respectively, which both cause defects in the MXene-K^+^ framework and result in PW flowing away in molten state. Evidently, minimal leakage was found under MK2@PW and MK3@PW at 85 °C, and the PW mass loading ratios of MK2@PW, MK3@PW still retained 92.9 and 96.2 wt% (Fig. 2b), respectively. More to the point, benefiting from the reinforcement of moderate K^+^ on the framework, no obvious leakage could be observed under the MK3@PW PCCs even with an 80 g weight at 85 °C, which is more than 160 times higher than that of the sample. This result is consistent with the compression resistance of MXene-K^+^ aerogel (Fig. S4). To further explore the leakage mechanism of the PCCs, the surface of PCCs (the PCCs that had been heated on filter paper for 12 h) was captured by SEM. As displayed in Fig. 2c, skeleton of MXene@PW was almost destroyed by volume expansion and contraction of PW during their melting and crystallization. Conversely, the frameworks of MXene- K^+^@PW PCCs were preserved completely (Fig. 2d–g) due to the electrostatic coordination interactions between K^+^ and the –OH, and –F bonds of MXene nanosheets. In particular, when the mass ratio of MXene to KCl was 1:2 to 1:4, the surface of MK2@PW and MK3@PW showed a compact surface with no notch caused by PW leakage. Surprisingly, the loading of PW significantly improved the hydrophobicity of MXene surface, MK3@PW exhibited a water contact angle of 125° ± 2°, which is beneficial for protecting MXene from potential oxidation in humid environment. The above results revealed that this layered porous framework constructed by MXene nanosheets possesses the great packaging performance at high temperature and pressure, while also showing that the introduction of PW is conducive to broadening the application range of MXene-based composites.

### Thermophysical Properties and Thermal Reliability of MXene-KCl@PW PCCs

**Fig. 3:**
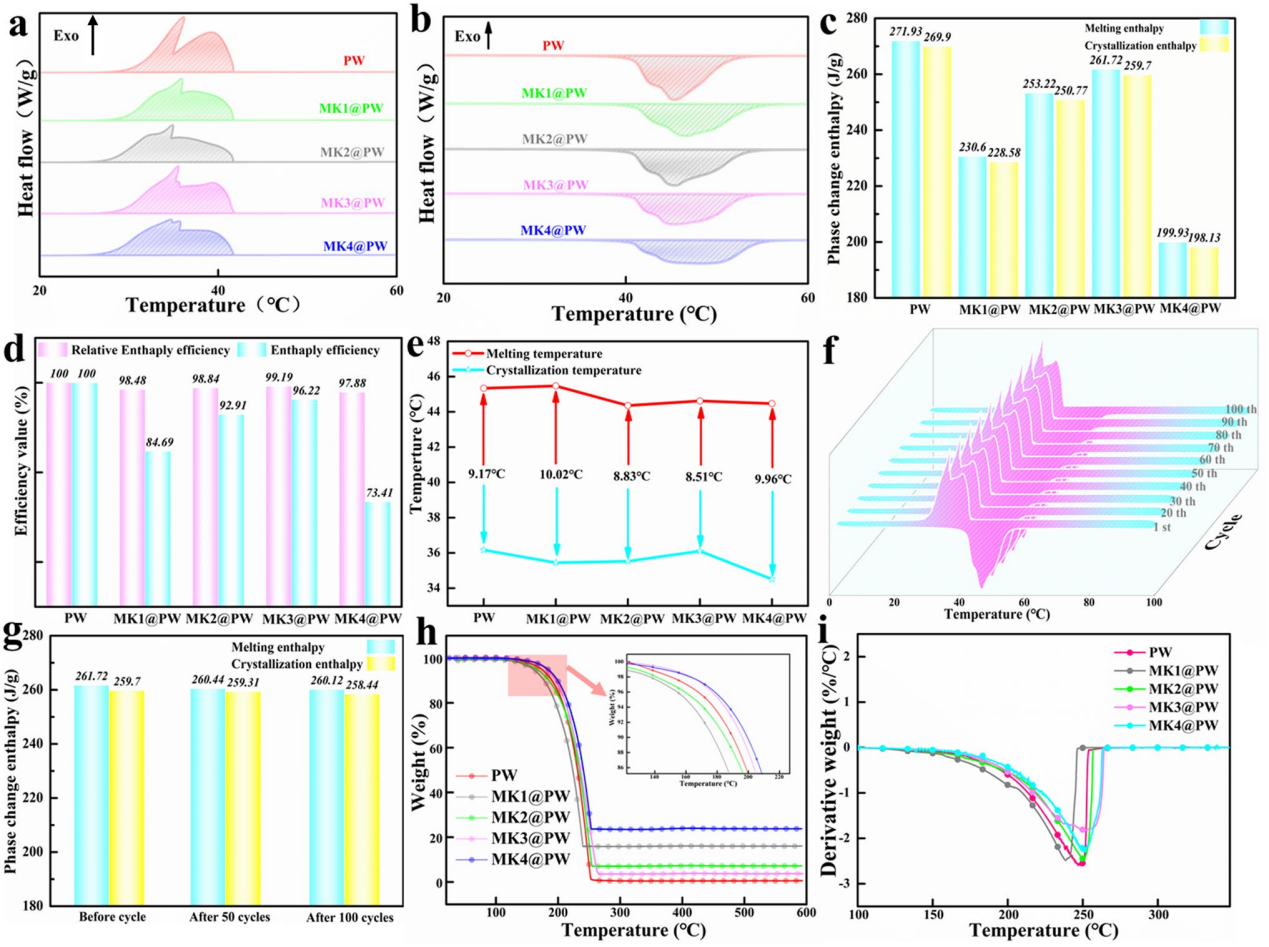
DSC curves of **a** exothermic and **b** endothermic processes of PW and different K^+^ content MXene-K^+^@PW; **c** phase change enthalpy value, **d** relative enthalpy efficiency and enthalpy efficiency, **e** melting temperature and crystallization temperature of PW and different K^+^ content MXene-K^+^@PW. **f** DSC curves and **g** phase change enthalpy value of MK3@PW after 100 times thermal cycles. **h** TG curves and **i** DTG curves of PW and different K^+^ content MXene-K^+^@PW

The thermophysical behaviors of PW and PCCs was tested by DSC measurement. The endothermic and exothermic effects of pure PW and MXene-K^+^@PW PCCs are presented in Fig. 3a, b, and corresponding phase transition parameters are listed in Table S1. As displayed in Fig. 3a, b, MXene-K^+^@PW PCCs showed distinct melting and crystallization behaviors, which are close to pure PW, whereas the crystallization temperature of PCCs lag slightly behind PW, which was due to the capillary force of aerogel slightly inhibits the crystallization behavior [40, 41] of PW molecule chains. Additionally, the Fourier transform infrared spectroscopy (FTIR) (Fig. S5) implied the encapsulation effect of aerogel is independent of chemical reaction. Figure 3c displays that crystallization and melting enthalpy of MK3@PW attached 261.7 and 259.7 J g^−1^, respectively, which is closest to PW. Benefiting from the ideal frameworks cross-linked by K^+^ and MXene nanosheets, the enthalpy efficiencies (λ) (Eq. S1) of MK2@PW and MK3@PW reached 92.91%, 98.84% (Fig. 3d), and their relative enthalpy efficiency (η) (Eq. S2) reached 96.22%, 99.19% (Fig. 3d), respectively. Besides, the difference between the melting and crystallization temperature of MXene-K^+^@PW is similar to pure PW (Fig. 3e). Those results suggest that MK2 and MK3 aerogel are extremely ideal supporters of PW, MK2@PW, and MK3@PW possess the ultrahigh energy storage density, their energy storage density, and enthalpy efficiency are higher than other aerogel-based PCCs (Table S2).

PCCs usually operate repeatedly in actual service life, for this, DSC and TG were used to investigate their durability and phase transition behavior after a long time of working, and these results are displayed in Fig. 3f–i. In Fig. 3f, the DSC curves after 100 cycles almost overlapped with the original curve, the melting and crystallization enthalpy after 100 cycles were 260.12 and 258.44 J g^−1^ (Fig. 3g), there are only 0.61% and 0.49% losses. Such negligible loss suggest that the MK3@PW possess a high reliability in TES. In addition, as shown in Fig. 3h, i, in contrast to pure PW, the thermal decomposition curve of MK3@PW is closed to a higher temperature. When the weight losses of PW attach 15%, the thermal decomposition temperature of MK3@PW attaches 205.8 °C, which is 5.6 °C higher than pure PW. This gain can be observed intuitively in the DTG curves (Fig. 3i), the maximum decomposition temperature decomposition temperature in MK3@PW is 252.1 °C. Pure PW is 245.4 °C, indicating that the introduction of MXene-K^+^ supporting skeletons enhance the thermal stability of PW. What’s more, no weight loss can be observed within the range of 100 °C around the phase change temperature of PW, suggesting that MK3@PW holds wide adaptability to operating temperatures. The above results imply that MK3@PW PCC provide substantial reliability for application in actual operating environment.

### Solar–Thermal–Electric Performance

**Fig. 4:**
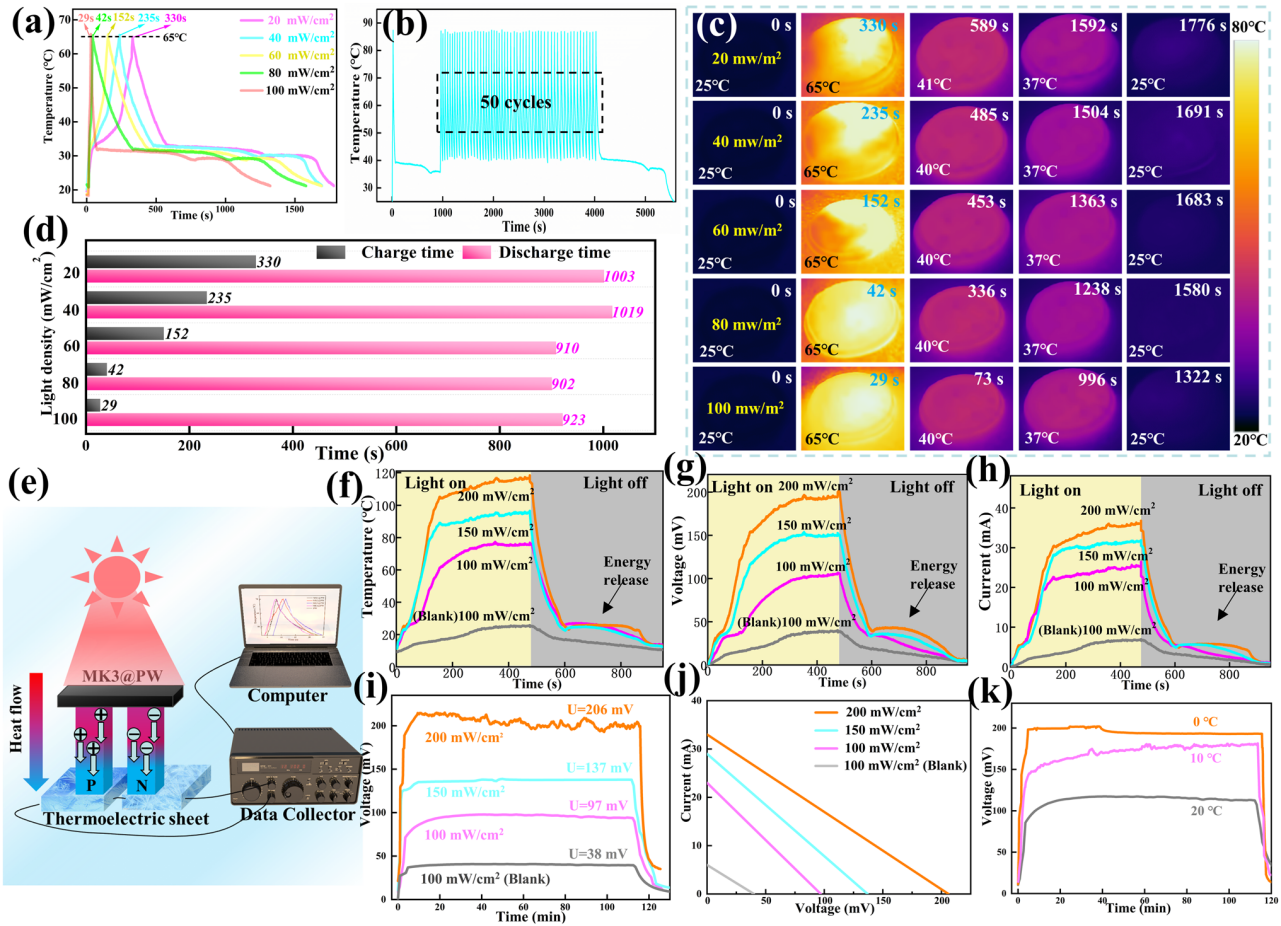
**a** Temperature–time curves; **b** Temperature–time curves of MK3@PW PCC after 50 cycles solar–thermal conversion. **c** Infrared thermogram and **d** charging and discharging time of MK1@PW, MK2@PW, MK3@PW, MK4@PW under different light density. **e** Schematic of a self-built solar–thermal–electricity platform. **f** Surface temperature, **g** the generated open-circuit voltage and **h** current of MK3@PW PCC under various light densities; **i** Voltage–time curves for 120 min and **j** corresponding current–voltage curves of MK3@PW PCC under different light densities; **k** Generated open-circuit voltage of MK3@PW PCC under different temperature of cold side

The solar–thermal conversion ability of MK3@PW PCC was tested using a homemade equipment (Fig. S6) under a stable simulation solar source. Temperature–time curves and corresponding IR thermography images are shown in Fig. 4a, c, respectively. With increasing light density, the temperature of MK3@PW PCC visibly increased (Fig. 4a, c). As the surface temperature of MK3@PW reached 65 °C, it took 330, 235, 152, 42, and 29 s under the light densities of 20, 40, 60, 80, and 100 mW cm^−2^, respectively, indicating that the layered MXene nanosheets frameworks display excellent light absorption. The surface temperature of MK3@PW decreased swiftly and later displayed a long-term constant temperature plateau even when light radiation was withdrawn, which demonstrates that thermal energy saved by MK3@PW PCC was released. Interestingly, in Fig. 4d, MK3@PW PCC can be maintained for 923 s in the range of 37–40 °C after light charging (100 mW m^−2^) for 29 s, indicating that the MK3@PW possesses a high-speed solar–thermal response and ultrahigh energy storage density. The calculated solar–thermal conversion efficiencies (Eq. S3) of MK3@PW under ascending light densities are 98.4%, 48.4%, 36.1%, 26.6%, and 21.4% (Table S3), respectively. Such outstanding solar-to-thermal conversion ability of MK3@PW PCC offers the potential for extensive applications in supplying heat to protect the human body and electronic equipment from cold environments.

To further improve the utilization of the heat absorbed and stored by MK3@PW from light, a thermoelectric sheet was sandwiched between the MK3@PW sample and thermostatic table (Fig. 4e), which can convert temperature differences into electrical voltages via Seebeck effect [42]. For comparison, the thermoelectric sheets without PCC covered was selected as the blank one. The temperature of MK3@PW reached 67 °C under 100 mW cm^−2^ (Fig. 4f), which is 45 °C higher than that of the blank one, suggesting that the MK3@PW can significantly improve the temperature on the hot side of thermoelectric devices. In addition, the hot side temperature furtherly increased to 115 °C under a higher light density at 200 mW cm^−2^. At the same time, due to the greater differences in temperature make for the higher power generation capacity of thermoelectric devices [43], the output voltage, current, and power of MK3@PW-loaded thermoelectric device are about 101 mV, 24 mA, 2.424 mW respectively, which are improved by 158%, 267%, 850% than the blank. More importantly, when the light was cutoff, an obvious holding of the current and voltage can be found, it results from the thermal energy saved in MK3@PW PCC have extended the power generation. This means that even under the condition of no illumination, it can continue to generate continuous voltage and current due to phase change energy storage. Detailly, for this lasting voltage and current, the average voltage and current generated by MK3@PW-loaded thermoelectric devices are 37.2 mV and 6.5 mA, while those of blank are only 5.2 mV and 1.2 mA, respectively. The power generation based on pure phase changing provided the MK3@PW PCC a broader application in utilization of solar energy.

In addition, a test under different light and different cold source temperatures for 120 min was applied to assess the long-term working constancy of MK3@PW PCC. As shown in Fig. 4i–k, a, long stable voltage platform can be observed, whether under high light intensity or different cold junction temperatures, and a greater voltage will be generated with the increase of light density and the decrease of cold source. When the light density was 150 mW cm^−2^, the temperature of cold side of the thermoelectric devices were set at 20, 10, and 0 °C, respectively, corresponding to open-circuit voltages of 197, 180, and 117 mV (Fig. 4k), respectively. The excellent generation performance, stable output capacity, especially the continuous endurance without light radiation, presents a new strategy for achieving high efficiency in practical application using solar energy.

### Electric/Magnetic–Thermal Conversion of MXene-K^+^@PW PCCs

**Fig. 5:**
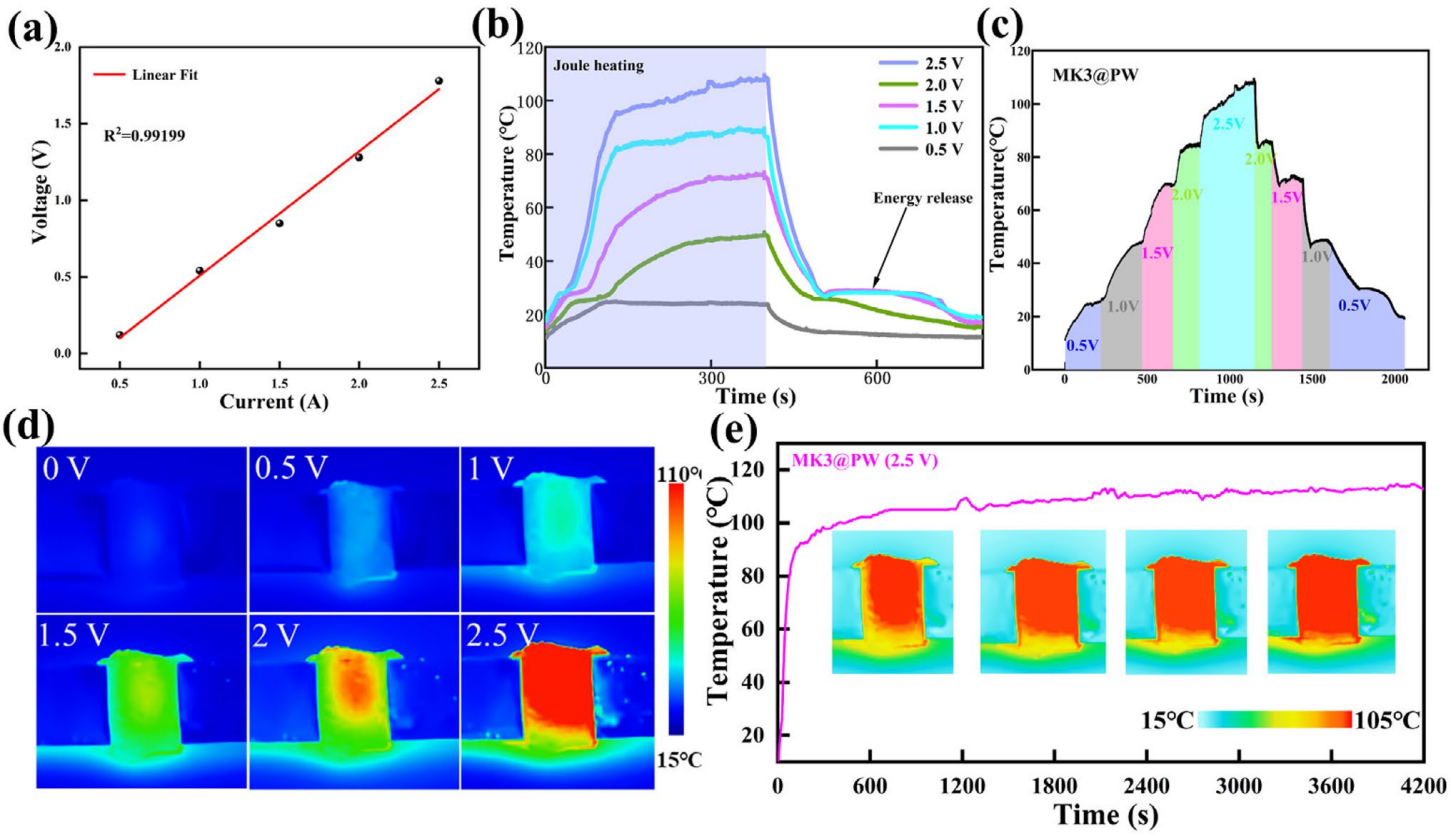
**a** Voltage-current curves of MK3@PW PCC; **b** surface temperature of MK3@PW PCC under different applied voltages. **c** Temperature evolution under stepwise-increased/decreased and **d** corresponding IR thermal imagers of MK3@PW PCC; **e** surface temperature under long-time electric-thermal operation

Nevertheless, the source of solar energy is uncontrollable, making it essential to develop the controllable energy to also achieve more stable thermal energy conversion. Electric energy is a favorable candidate as a conversion source of heat energy due to the Joule effect [44, 45]. As shown in Fig. 5a, the change in voltage and current of MK3@PW is almost proportional (fitting degree reaches 0.99199), suggesting that the electrical heating process of MK3@PW follows the Joule effect. The temperature–time curves and corresponding IR images of MK3@PW under different external voltages (0.5–2.5 V) are displayed in Fig. 5b, e. The saturation temperature of MK3@PW reaches 24.4, 47.8, 70.6, 87.4, and 105.4 °C, respectively. This excellent electrothermal conversion capability is attributed to the complete network structure of the MXene-K^+^ aerogel. Moreover, after finishing the process of phase transition, the constant temperature can be maintained long for 210 s, such ability will provide a comfortable temperature environment for the human body and electronic devices for a long time. Moreover, timely responsiveness of MK3@PW PCC under different driving voltages furtherly expands the applied range of the actual environment (Fig. 5c). To explore the stability of MK3@PW during the electrothermal conversion process, MK3@PW demonstrates stable electrothermal conversion capability at 2.5 V, the IR images of MK3@PW at 800, 1,700, 2,600, and 3,600 s have almost no variation, implying that the MK3@PW PCC holds reliable dependability even the environment temperature is above 100 °C.

**Fig. 6:**
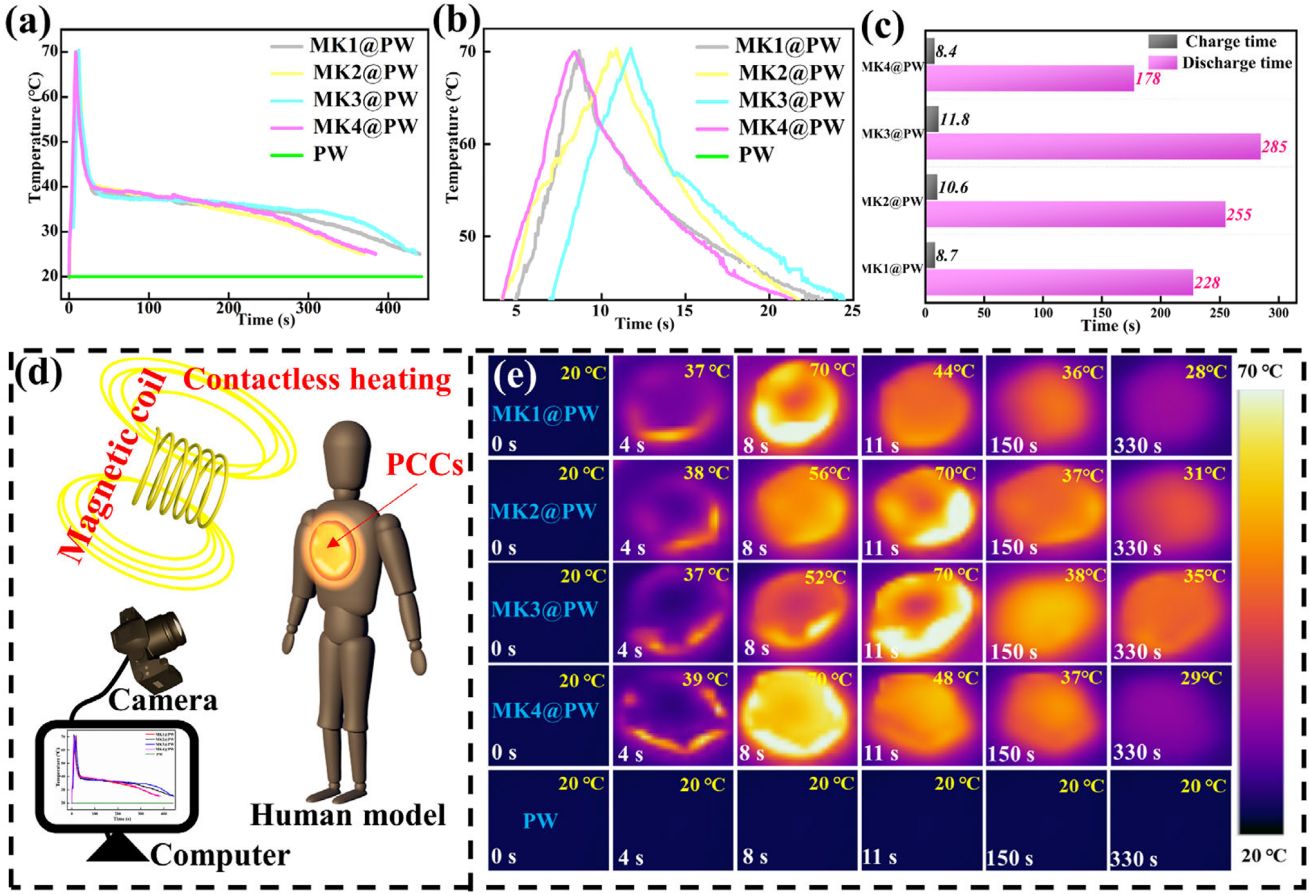
**a**, **b** magnetic–thermal conversion curves, **c** magnetic–thermal charging time and discharging time and **e** corresponding IR thermal images of different K^+^ content MXene-K^+^@PW; **d** magnetic thermotherapy for human model

Magnetic energy is another man-made controllable energy source that can be converted into thermal energy. In this conversion process, magnetic materials can generate much heat in the alternating magnetic field provided by periodic electron motion due to Neel relaxation or Brownian relaxation [46, 47]. Since the magnetic field does not need to be in direct contact with the human body, it can also be used to serve thermal therapy for the human body. Therefore, the magnetic–thermal conversion ability of MK3@PW PCC was explored by a magnetic field device. As displayed in Fig. 6c, a, coil is placed above the PCC-loaded human model, and the corresponding temperature–time curve and infrared thermal imager is recorded in Fig. 6a, b, e, respectively. As shown in Fig. 6a, b, all the MXene-K^+^@PW PCCs exhibited similar magnetic–thermal conversion abilities, and MK1@PW, MK2@PW, MK3@PW, and MK4@PW took 8.7, 10.6, 11.8, and 8.4 s to reach 70 °C, respectively, the variance in times comes from the difference in the enthalpy of PCCs, corresponding phase transition time is 228, 285, 255, and 178 s, respectively. This controllable, rapid, and noncontact charging thermal energy conversion path points out a new strategy for the utilization of MXene-based PCCs.

### Electromagnetic Shielding Performance of MXene-KCl@PW PCCs

**Fig. 7:**
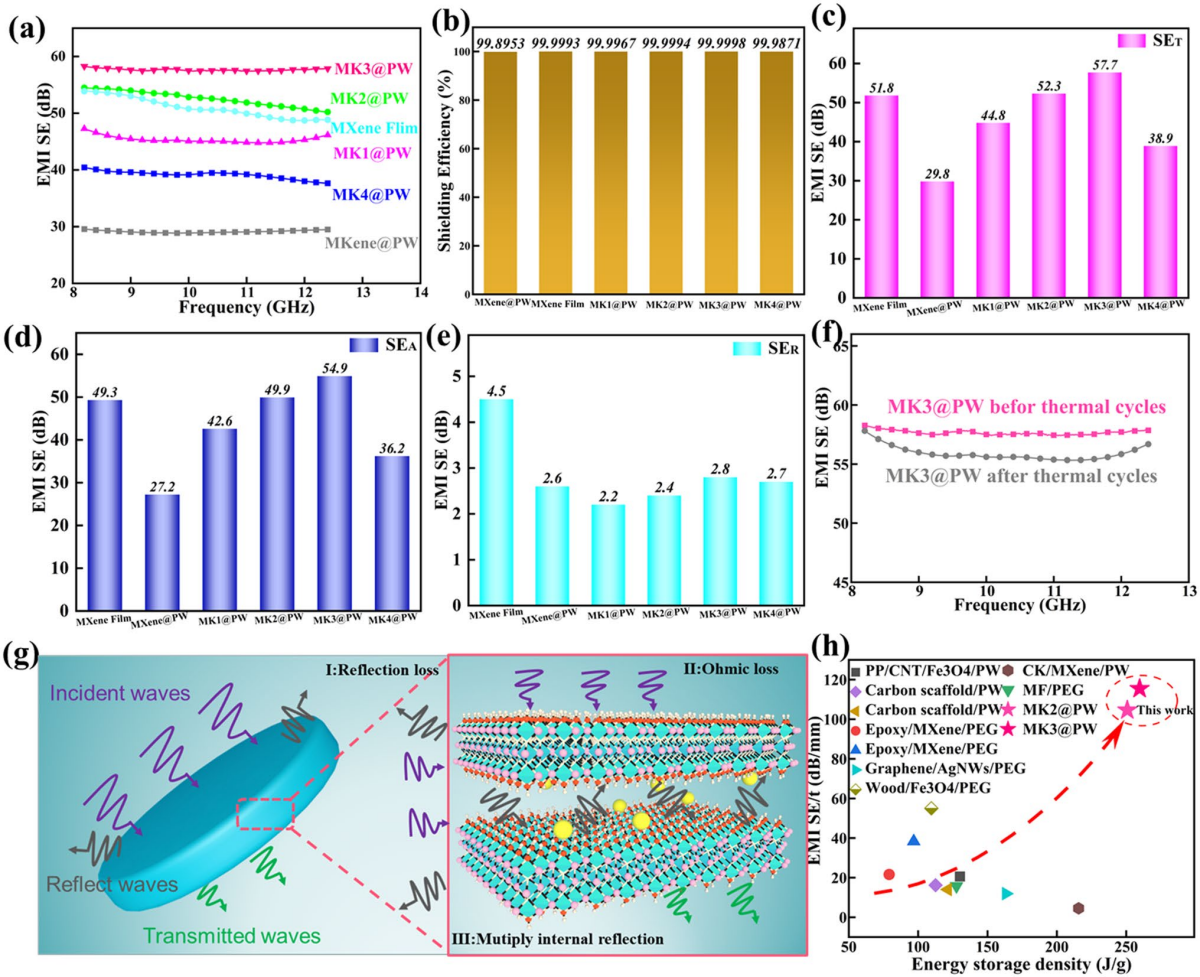
**a** The EMI SE curves, **b** shielding efficiencies and the average **c** SE_T_, **d** SE_A_ and **e** SE_R_ values of MXene film, MXene@PW, MK1@PW, MK2@PW, MK3@PW and MK4@PW PCCs. **f** EMI SE curves before and after 100 thermal cycles. **g** EMI shielding mechanism of the MXene-K^+^@PW PCCs. **h** Comparison of EMI SE/t (dB mm^−1^) and energy storage density (J g^−1^) for different PCCs

With the popularity of wireless networks and small electronic equipment, massive EMWs (electromagnetic waves) generated, resulted in critical damage to devices and mankind health [48, 49]. In order to further protect human bodies and electronic devices from electromagnetic pollution except thermal management capability discussed above, electromagnetic shielding capability of MXene-K^+^@PW PCCs also was discussed. Figure 7a displays that the EMI shielding performance of MXene-K^+^@PW has a weak dependence on frequency (8.2–12.4 GHz). Among them, MK3@PW PCC exhibited the highest SE value (57. 7 dB), while the SE value of MXene film and MXene@PW with the same mass of MXene are only 51.8 and 29.8 dB, respectively. Moreover, the EMI SE of MK1@PW and MK4@PW were lower than that of MK3@PW because of insufficient and excessive K^+^ lead to obvious fracture holes and defects, which caused the leakage of EMWs. Note that the MK3@PW can exhibit a remarkable EMI SE 57.7 dB (significantly greater than commercial standards of 20 dB), which can inhibit 99.9998% of the incident EMWs (Fig. 7b). Figure 7c–e exhibits the total EMI shielding effectiveness (SE_T_), microwave refection (SE_R_), and microwave absorption (SE_A_) of these samples computed by Eqs. S4-S9. With the increasing K^+^, the MK2@PW and MK3@PW PCCs exhibit significantly increased SE_T_ and SE_A_ with mildly increased SE_R_. It is that the SE_A_ accounts for the majority of SE_T_ mostly in most of the nanomaterials, showing that the EMI shielding performances are mainly dominated by the absorption of electromagnetic waves [50]. Furthermore, the SE value after 100 times thermal cycles can reach 98% of the original value (Fig. 7f), indicating the stable electromagnetic shielding performance of MK3@PW PCC through multiple phasing changing cycles. To further understand the processes of electromagnetic shielding, the transfer path of EMW in the MXene-K^+^@PW is displayed in Fig. 7g. As EMW come into contact with the top of MXene-K^+^@PW PCCs, some incident EMWs immediately reflects as a result of the discontinuity in impedance from atmosphere to MXene nanosheets. Then, vast majority of the EMWs access the charge carriers of MXene-K^+^ conductive network, making for loads of ohmic losses and the attenuated EMW was converted to heat loss [51, 52]. In addition, multiple internal reflections in the layered channels of MXene-K^+^@PW PCCs as well as make the contribution for the dissipation of EMWs [53, 54], therefore making for the improving whole electromagnetic shielding efficiency. Benefitting from the ordered arrangement of MXene nanosheets and the strengthening of MXene aerogel by K^+^, both of SSE/t and energy storage density of MK2@PW and MK3@PW PCCs exceed great majority of the composites reported in previous works listed in Table S4 and Fig. 7h.

## Conclusions

In summary, a series of multifunctional MXene/K^+^/PW PCCs with high solar–thermal conversion efficiency, solar–thermal–electric conversion, electric–thermal conversion, magnetic–thermal conversion and excellent EMI efficiency properties were successfully prepared via vacuum-assisted filtration and vacuum impregnation process. First, the high porosity of MXene-K^+^ aerogel realizes the ultrahigh mass loading (97.1 wt%) of PW in MK3@PW PCCs, as a consequence, MK3@PW achieves an extremely high energy density (melting enthalpy value (261.72 J g^−1^), crystallization enthalpy value (259.70 J g^−1^)). Then, due to the absence adhesives compared with traditional MXene-based aerogel, the MK3@PW exhibits a high light-to-thermal conversion efficiency (98.4%), notably, MK3@PW can further convert the collected heat energy into electric energy through thermoelectric equipment and realize favorable solar–thermal−electric conversion (producing 206 mV of voltage with light radiation intensity at 200 mW cm^−2^). An excellent joule heat performance (reaching 105 °C of temperature with the input voltage of 2.5 V) and good magnetic–thermal conversion behavior for contactless thermotherapy also possessed by MK3@PW. Besides, especially, as a result of ordered arrangement of MXene nanosheets, MK3@PW PCC exhibits higher electromagnetic shielding efficiency value (57.7 dB) than MXene@PW (29.8 dB) with same mass of MXene. In brief, this work proposes a new opportunity for the multi-scene response and practical application of PCMs to satisfy the demand for next-generation multifunctional PCCs.

## Supplementary Information

Below is the link to the electronic supplementary material.

Supplementary file1 (DOCX 2059 KB)
