# Correction: Self-Assembly of Binderless MXene Aerogel for Multiple-Scenario and Responsive Phase Change Composites with Ultrahigh Thermal Energy Storage Density and Exceptional Electromagnetic Interference Shielding

**DOI:** 10.1007/s40820-024-01582-3

**Published:** 2025-01-15

**Authors:** Chuanbiao Zhu, Yurong Hao, Hao Wu, Mengni Chen, Bingqing Quan, Shuang Liu, Xinpeng Hu, Shilong Liu, Qinghong Ji, Xiang Lu, Jinping Qu

**Affiliations:** 1 Key Laboratory of Material Chemistry for Energy Conversion and Storage of Ministry of Education, School of Chemistry and Chemical Engineering, Huazhong University of Science and Technology, Wuhan, 430074 People’s Republic of China; 2 Qingdao Mental Health Center, Qingdao, 266034 People’s Republic of China; 3 Hubei Engineering Research Center for Biomaterials and Medical Protective Materials, Huazhong University of Science and Technology, Wuhan, 430074 People’s Republic of China; 4 Hubei Key Laboratory of Material Chemistry and Service Failure, School of Chemistry and Chemical Engineering, Huazhong University of Science and Technology, Wuhan, 430074 People’s Republic of China; 5 National Engineering Research Center of Novel Equipment for Polymer Processing, Key Laboratory of Polymer Processing Engineering (South China University of Technology), Ministry of Education, Guangdong Provincial Key Laboratory of Technique and Equipment for Macromolecular Advanced Manufacturing, School of Mechanical and Automotive Engineering, South China University of Technology, Guangzhou, 510641 People’s Republic of China

**Correction to: Nano-Micro Lett. (2024) 16:57** 10.1007/s40820-023-01288-y.

Following publication of the original article [1], the authors reported that the order of the images in Figs. 5 and 6 were reversed, the positions of the images needed to be exchanged. Another mistake is that the author inadvertently copied the same image in Fig. 2(f) with Fig. 2(e).

The correct Figs. 2, 5 and 6 have been provided in this correction.

The original article [1] has been updated.

The incorrect Fig. 2 is:

**Fig. 2:**
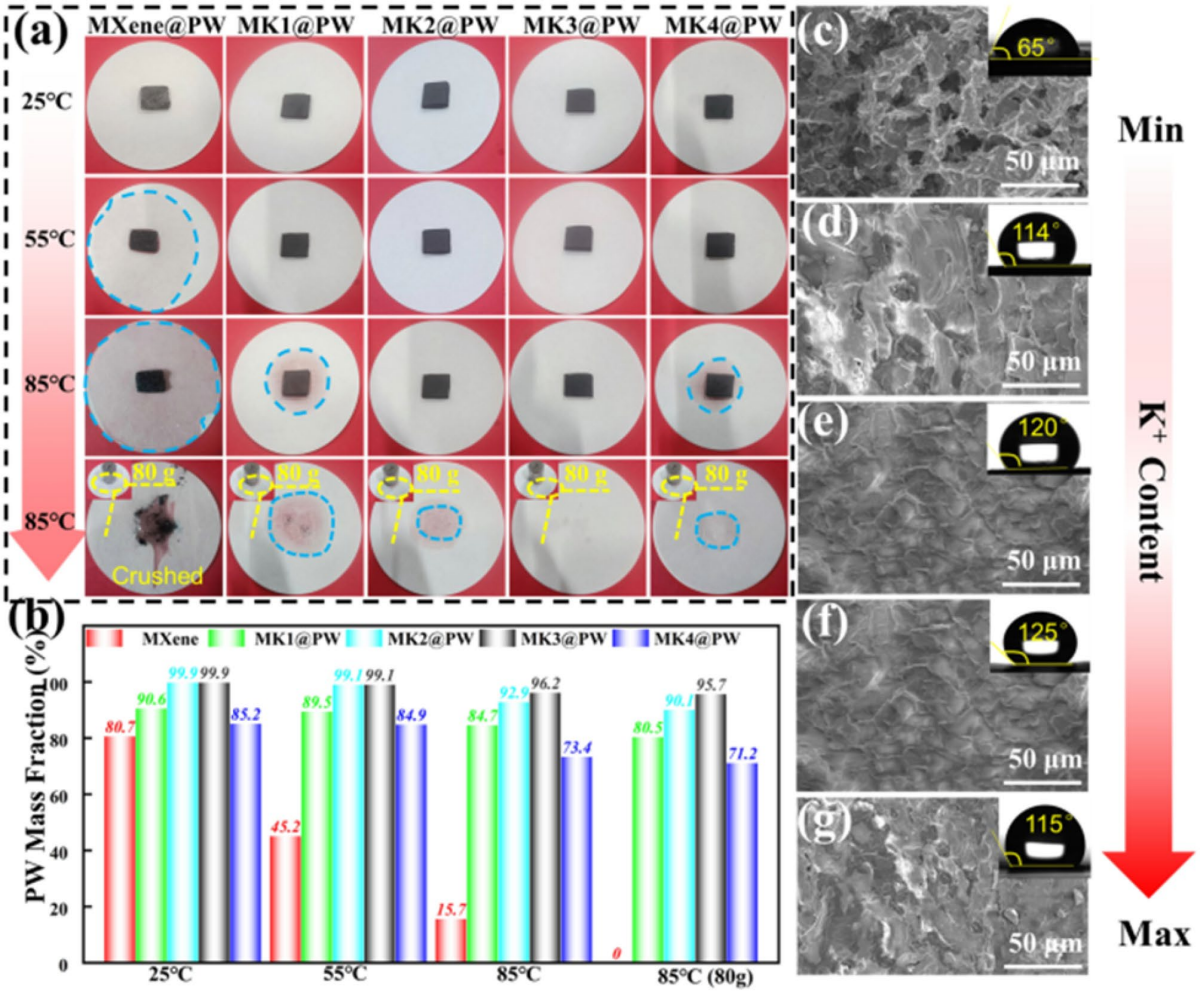
**a** Leakage test of MXene@PW, MK1@PW, MK2@PW, MK3@PW, MK4@PW under different temperatures and pressures; **b** PW mass loading after different temperature and pressure leakage test; upper surface SEM images and water contact angle of **c** MXene@PW, **d** MK1@PW, **e** MK2@PW, **f** MK3@PW, **g** MK4@PW after leakage test under 85 °C for 10 h

The correct Fig. 2 is:

**Fig. 2:**
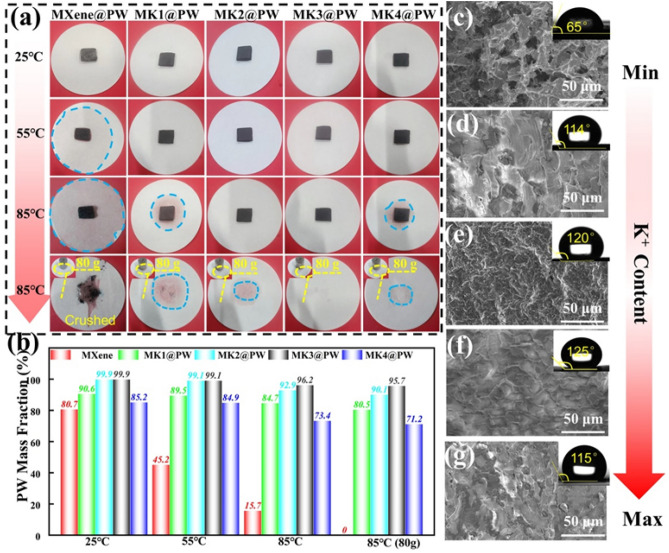
**a** Leakage test of MXene@PW, MK1@PW, MK2@PW, MK3@PW, MK4@PW under different temperatures and pressures; **b** PW mass loading after different temperature and pressure leakage test; upper surface SEM images and water contact angle of **c** MXene@PW, **d** MK1@PW, **e** MK2@PW, **f** MK3@PW, **g** MK4@PW after leakage test under 85 °C for 10 h

The incorrect Fig. 5 is:

**Fig. 5:**
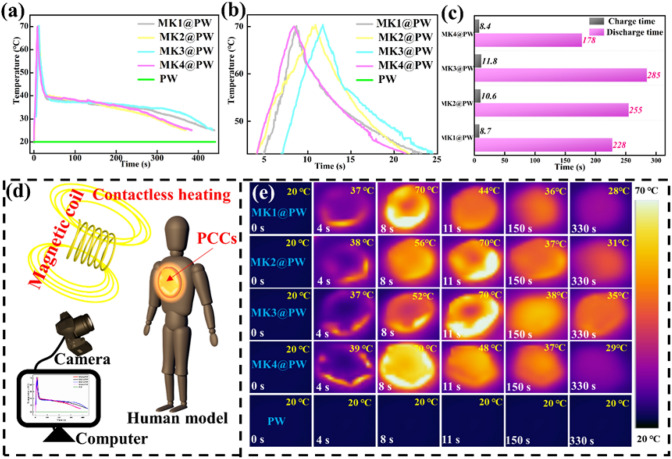
**a** Voltage–current curves of MK3@PW PCC; **b** surface temperature of MK3@PW PCC under different applied voltages. **c** Temperature evolution under stepwise-increased/decreased and **d** corresponding IR thermal imagers of MK3@PW PCC; (**e**) surface temperature under longtime electric-thermal operation

The correct Fig. 5 is:

**Fig. 5:**
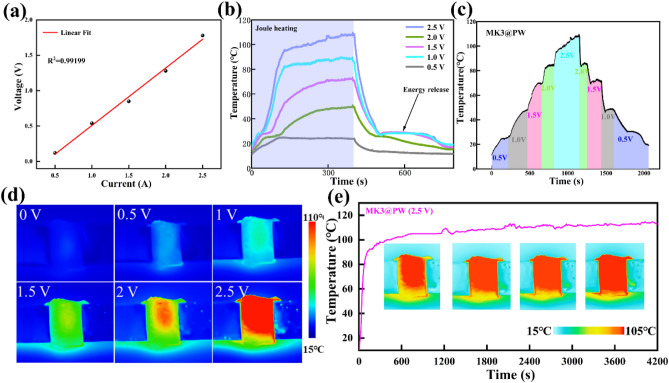
**a** Voltage–current curves of MK3@PW PCC; **b** surface temperature of MK3@PW PCC under different applied voltages. **c** Temperature evolution under stepwise-increased/decreased and **d** corresponding IR thermal imagers of MK3@PW PCC; **e** surface temperature under longtime electric-thermal operation

The incorrect Fig. 6 is:

**Fig. 6:**
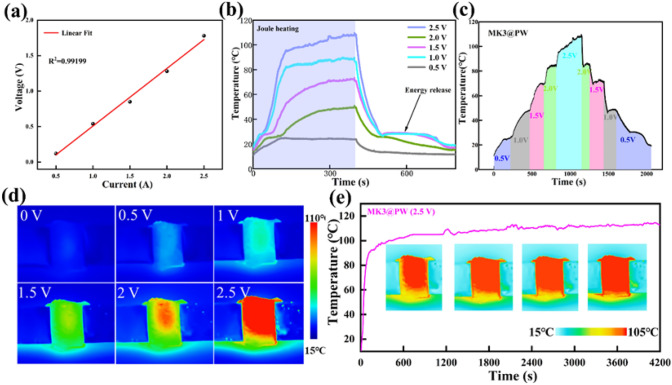
**a, b** Magnetic–thermal conversion curves, **c** magnetic–thermal charging time and discharging time and **e** corresponding IR thermal images of different K^+^ content MXene-K^+^@PW; **d** magnetic thermotherapy for human model

The correct Fig. 6 is:

**Fig. 6:**
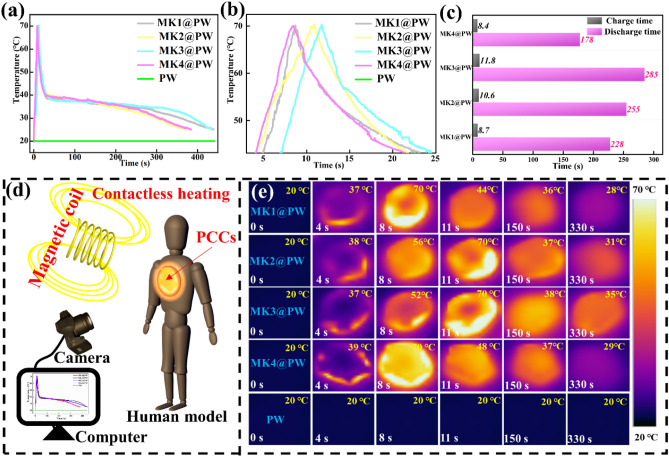
**a, b** Magnetic–thermal conversion curves, **c** magnetic–thermal charging time and discharging time and **e** corresponding IR thermal images of different K^+^ content MXene-K^+^@PW; **d** magnetic thermotherapy for human model

## References

[1] C. Zhu, Y. Hao, H. Wu et al., Self-assembly of binderless MXene aerogel for multiple-scenario and responsive phase change composites with ultrahigh thermal energy storage density and exceptional electromagnetic interference shielding. Nano-Micro Lett. **16**, 57 (2024). 10.1007/s40820-023-01288-y 10.1007/s40820-023-01288-y PMC10728427 38110610

