# Supplementary material for: Self-Assembly of Binderless MXene Aerogel for Multiple-Scenario and Responsive Phase Change Composites with Ultrahigh Thermal Energy Storage Density and Exceptional Electromagnetic Interference Shielding
Source: Nanomicro Lett. 2023 Dec 18;16:57. doi: 10.1007/s40820-023-01288-y (PMC10728427; doi:10.1007/s40820-023-01288-y)
Supplement: Supplementary file 1 — Supplementary file1 (DOCX 2059 KB) [file 40820_2023_1288_MOESM1_ESM.docx]

**Supporting Information**

**Self-Assembly of Binderless MXene Aerogel for Multiple-Scenario and Responsive Phase Change Composites with Ultrahigh Thermal Energy Storage Density and Exceptional Electromagnetic Interference Shielding**

Chuanbiao Zhu ^a, c, d^, Yurong Hao ^b^, Hao Wu ^a, c, d^ *, Mengni Chen ^a, c, d^, Bingqing Quan ^a, c, d^, Shuang Liu ^a, c, d^, Xinpeng Hu ^a, c, d^, Shilong Liu ^a, c, d^, Qinghong Ji ^a, c, d^, Xiang Lu ^a, c, d^ , Jinping Qu ^a, c, d, e^

^a^ Key Laboratory of Material Chemistry for Energy Conversion and Storage of Ministry of Education, School of Chemistry and Chemical Engineering, Huazhong University of Science & Technology, Wuhan, 430074, China

^b^ Qingdao Mental Health Center, Qingdao, 266034, China

^c^ Hubei Engineering Research Center for Biomaterials and Medical Protective Materials, Huazhong University of Science & Technology, Wuhan, 430074, China

^d^ Hubei Key Laboratory of Material Chemistry and Service Failure, School of Chemistry and Chemical Engineering, Huazhong University of Science & Technology, Wuhan, 430074, China

^e^ National Engineering Research Center of Novel Equipment for Polymer Processing, Key Laboratory of Polymer Processing Engineering (South China University of Technology), Ministry of Education, Guangdong Provincial Key Laboratory of Technique and Equipment for Macromolecular Advanced Manufacturing, School of Mechanical and Automotive Engineering, South China University of Technology, Guangzhou 510641, China.

**Summary.**

The following supplementary materials include description of and additional results.

**Section 1.** Materials, characterization details, equipment parameters, test methods and conditions.

**Section 2.** Analysis of MXene.

**Results**

Figure S1. The SEM image of cross section of pure MXene aerogel.

Figure S2. The density of pure MXene flim, MK1, MK2, MK3, MK4 aerogel.

Figure S3. Digital images of MK1 hydrogel, MK2 hydrogel, MK3 hydrogel, MK4 hydrogel.

Figure S4. Compression test of MK1, MK2, MK3, MK4 aerogel.

Figure S5. FTIR spectra of PW, MXene, MK1@PW, MK2@PW, MK3@PW, MK4@PW.

Figure S6. Schematic of a self-built solar-thermal platform.

Table S1. The phase change parameters of pure PW, MK1@PW, MK2@PW, MK3@PW, MK4@PW.

Table S2. Comparison of phase transformation parameters under different support materials

Table S3. Solar-thermal conversion efficiency calculation data sheet.

Eq. S1-S3. Enthalpy efficiency and relative enthalpy efficiency.

Eq. S4-S11. Electromagnetic shielding calculation parameters.

**Section 1.**

**Materials.** Paraffin Wax (PW), MAX powder (Ti_3_AlC_2_, 99.5%, particle size-400 mesh) was purchased from Nanjing Mingshan New Material Technology Co., Ltd. Lithium fluoride (LiF, 99.0%, AR), lithium chloride (LiCl), and hydrochloric acid (HCl, 37 wt%) were obtained from Shanghai Aladdin Biochemical Technology Co., Ltd.

**Characterization.** The exfoliated state and thickness of MXene nanosheets were obtained using an atomic force microscope (AFM, SPM-9700, Shimadzu, Japan). The microstructures of obtained composites were observed by field emission scanning electron microscope (FE-SEM, Hitachi, SU8010) at an acceleration voltage of 5 kV. The fourier-transform infrared spectroscopy (FTIR) was used to analyze the chemical structures with a Nicolet Is 5 spectrophotometer (USA) at a scanning number of 64 using the ATR scanning method, and the wavenumber range used for analysis was 500-4000 cm^-1^. X-ray photoelectron spectra (XPS, Thermo Scientific K-Alpha, USA) was employed to probe the chemical situations of MXene nanosheets. The crystalline behaviors were recorded by X-ray diffraction (XRD Rigaku Smart Lab-SE, Japan) using Cu-Kα radiation (40 kV, 30 mA) at room temperature, the diffraction angle range is 5°-65°, and the scanning speed was 10°/min. The phase change behaviors were tested by the differential scanning calorimeter (DSC, Waters, America), the rates of heating and cooling were 10 °C/min. The thermal stability was characterized by the thermogravimetric analyzer (TGA, Waters, America) in the range of 25℃-600℃ under N_2_ atmosphere. The thermal cycle testing was tested via the high-low temperature chamber (KSON KTHB-415TBS, China) between 0 and 100 °C with a heating and cooling rate of 5 °C/min. The solar-thermal conversion and thermal energy storage of the obtained PCCs were studied via simulated solar irradiation and the temperatures were documented using infrared thermography camera (Fotric 226s, Fotric Inc.). The power density of simulated solar was measured by Solar simulator (Beijing Zhongjiao Jinyuan Technology Co, Ltd., China.) Thermoelectric conversion was done through thermoelectric sheets (PCR025033, China), the cold source provided by the temperature chamber (KSON-KTHB-415TBS, China). The electro-thermal conversion and energy storage of the obtained PCCs were studied by a self-built circuit device. The output power source used in this work was DC power supply with switching mode (MS-305DS, Shenzhen Pioneer Instrument Technology Co. Ltd., China). The EMI shielding property of the samples was examined on a vector network analyzer (Agilent, PNA-N5244A, USA) over 8.2-12.4 GHz frequency range (in the X band) based on the waveguide method. The size of the test samples is a rectangle of 22.90×10.80 mm^2^.

**Section 2.**

**Analysis of MXene.**

**
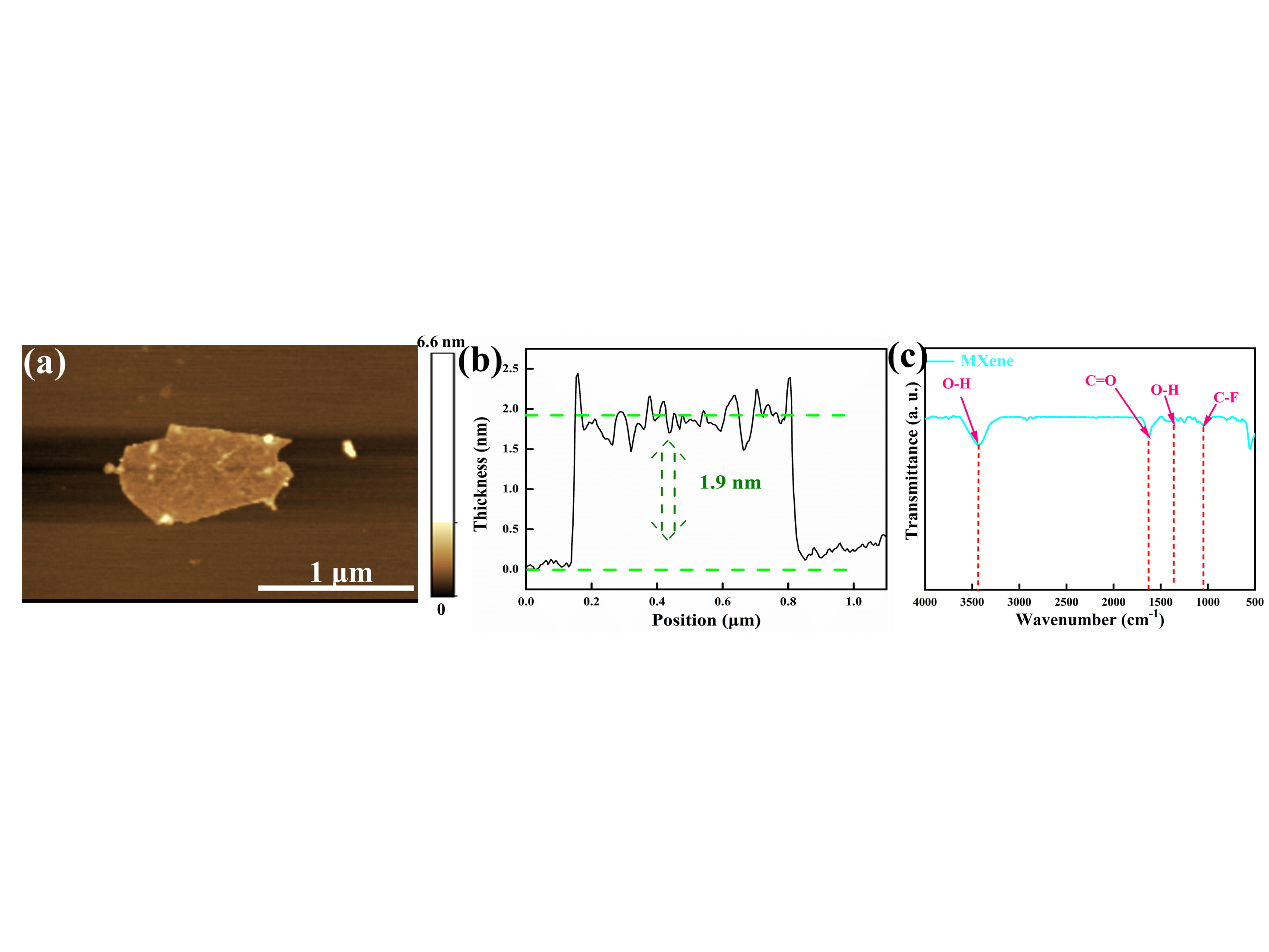
**

Figure 1 **(**a, b) AFM pictures of singled layered Ti_3_C_2_ MXene nanosheets and the corresponding height profile along the drawn lines. (c) FTIR spectra of Ti_3_C_2_ MXene.

Atomic force microscopy (AFM) was used to measure the morphology of MXene (Figure 1a). MXene shows the 2D structure with lateral dimensions of nanometers and average thickness of about 1.9 nm (Figure 1b). Fourier transform infrared (FTIR) spectrometer and X-ray diffraction (XRD) were applied to study the structure of MXene. As shown in Figure 1c, the stretching vibrations at around 3430, 1625, 1125 cm^-1^ in the FTIR spectrum are assigned to O-H, C=O, C-O, respectively, which are attributed to the large number of surface functional groups formed during the preparation process. Here, MXene has been successful prepared.

**Resluts.**


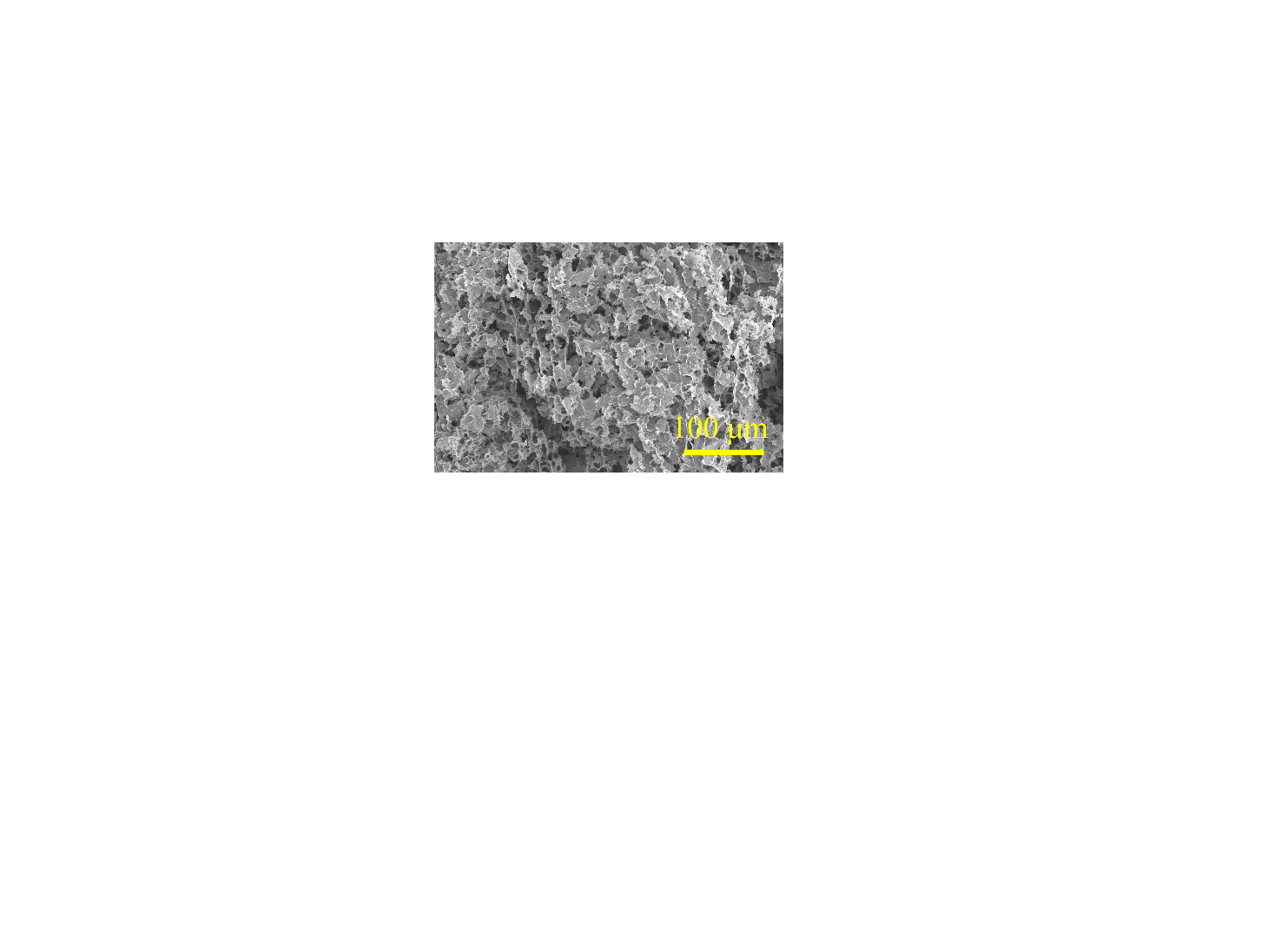


Figure S1. The SEM image of cross section of pure MXene aerogel.


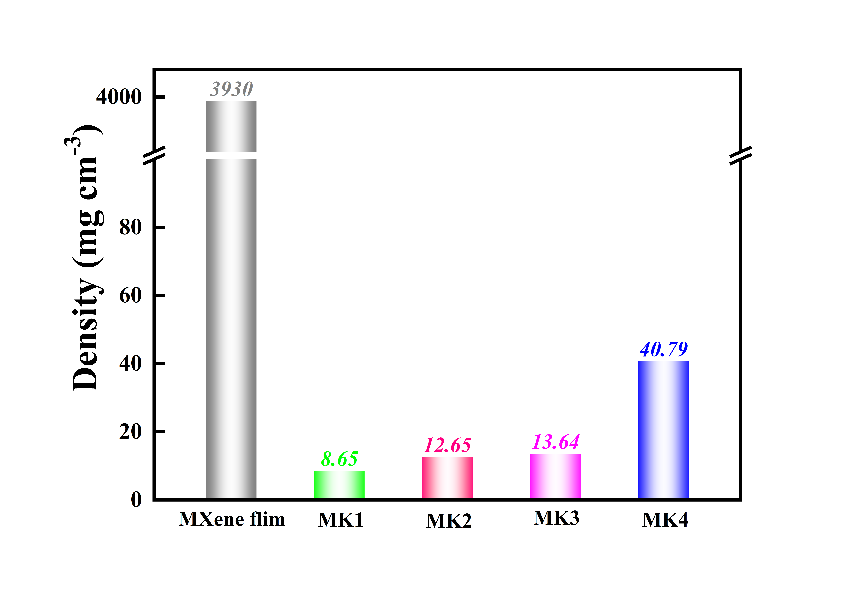


Figure S2. The density of pure MXene flim, MK1, MK2, MK3, MK4 aerogel.


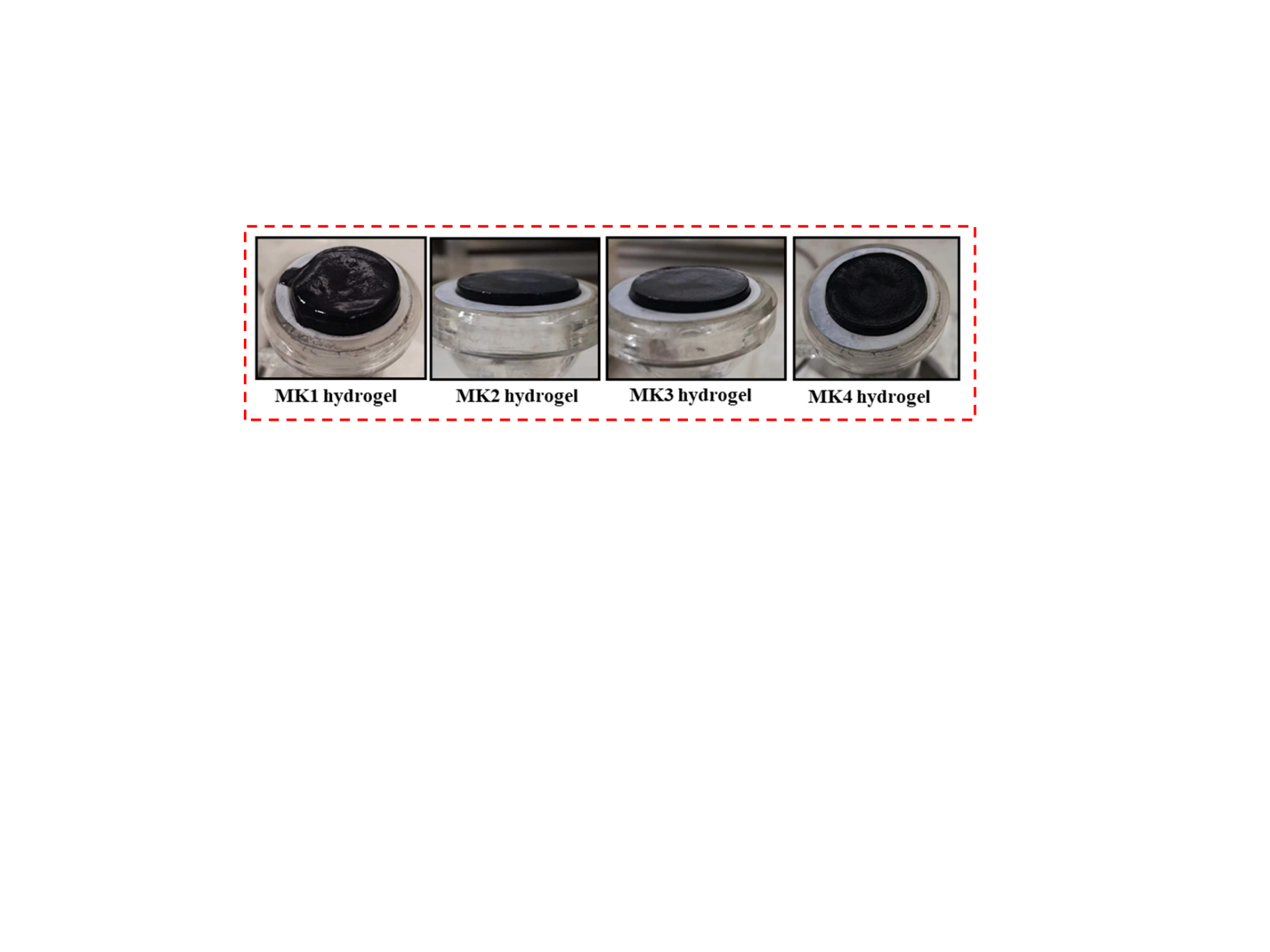


Figure S3. Digital images of MK1 hydrogel, MK2 hydrogel, MK3 hydrogel, MK4 hydrogel.


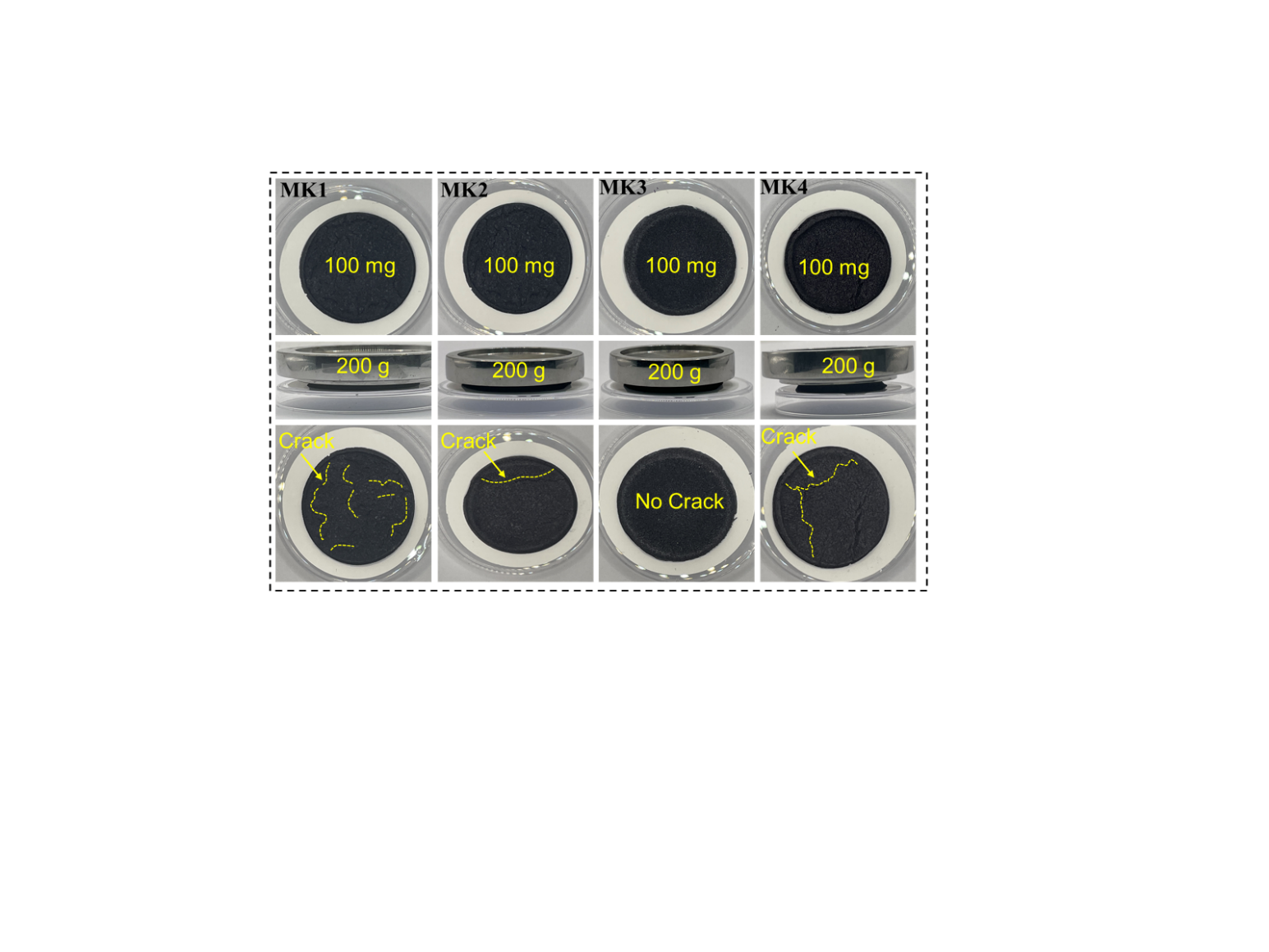


Figure S4. Compression test of MK1, MK2, MK3, MK4 aerogel.


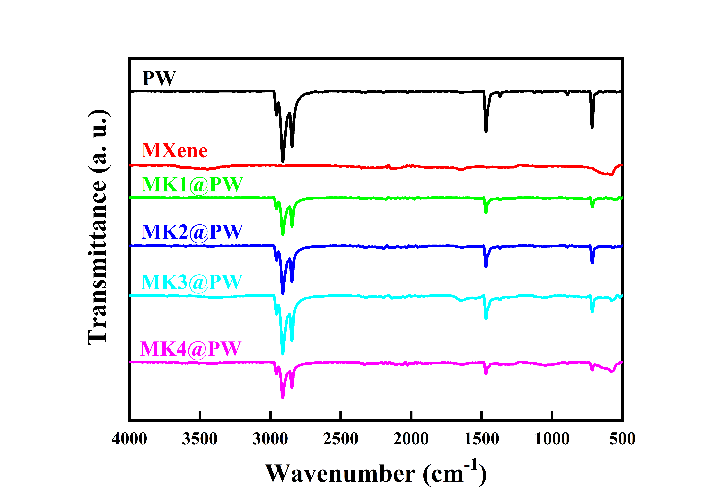


Figure S5. FTIR spectra of PW, MXene, MK1@PW, MK2@PW, MK3@PW, MK4@PW.


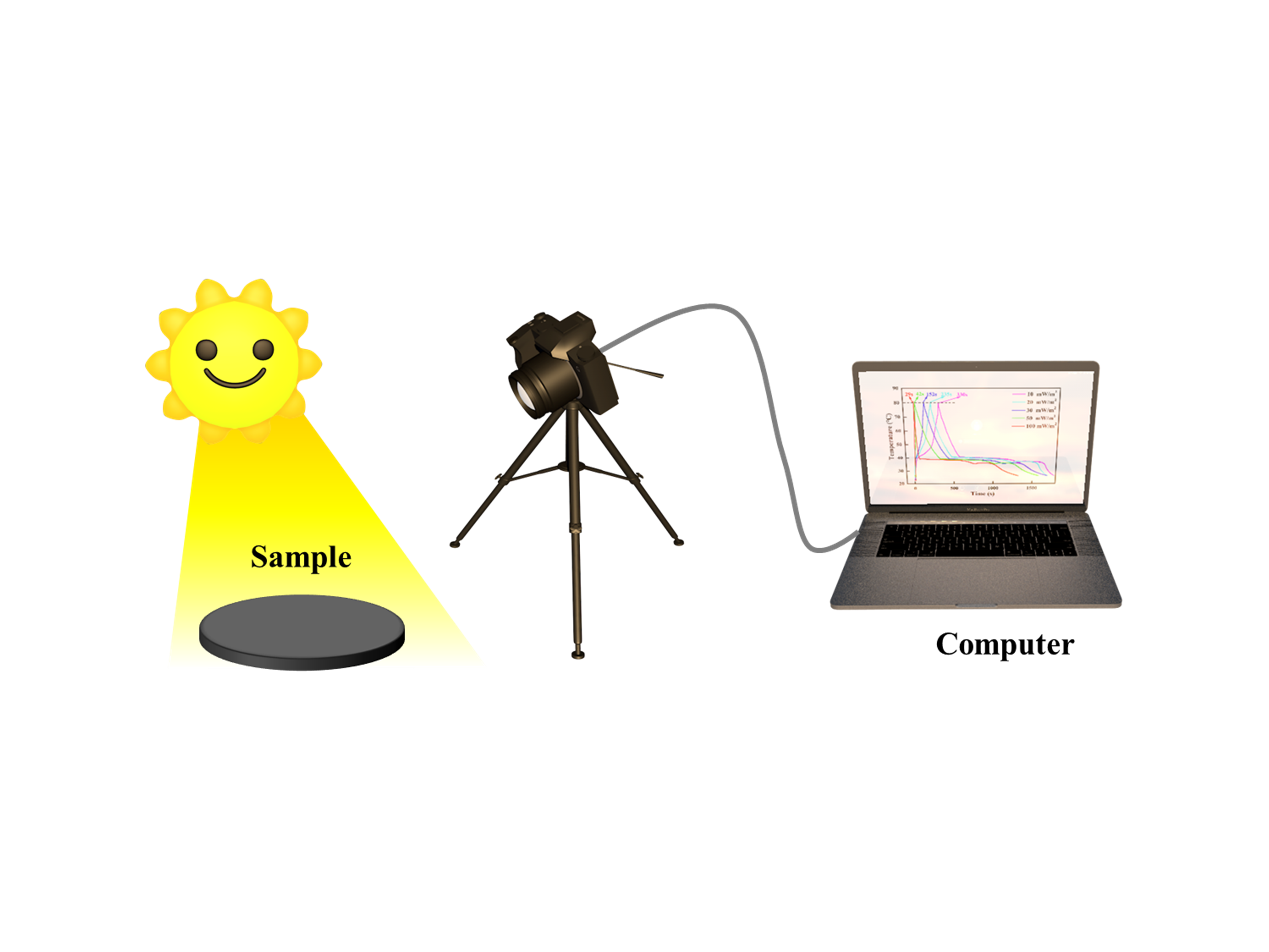


Figure S6. Schematic of a self-built solar-thermal platform.

**Table S1**. The phase change parameters of pure PW, MK1@PW, MK2@PW, MK3@PW, MK4@PW.

| Samples | T_mo_(°C) | T_mp_(°C) | T_me_(°C) | T_co_ (°C) | T_cp_ (°C) | T_ce_ (°C) |
| --- | --- | --- | --- | --- | --- | --- |
| PW | 40.52 | 45.34 | 51.87 | 41.67 | 36.17 | 31.70 |
| MK1@PW | 40.52 | 44.47 | 54.38 | 41.52 | 35.45 | 29.48 |
| MK2@PW | 35.47 | 45.36 | 53.88 | 41.48 | 35.53 | 29.75 |
| MK3@PW | 39.44 | 45.62 | 55.89 | 41.78 | 34.91 | 28.31 |
| MK4@PW | 40.86 | 44.47 | 54.69 | 41.04 | 34.51 | 28.71 |

T_mo_: onset melting temperature; T_mp_: peak melting temperature; T_me_: end melting temperature; T_co_: onset crystallization; T_cp_: peak crystallization temperature; T_ce_: end crystallization temperature.

**Table S2**. Comparison of phase transformation parameters under different support materials.

| Supporting materilas | PCMs | Melting  Point (℃) | Freezing  Point (℃) | Melting enthalpy（J/g） | Crystallization enthalpy（J/g） | Loading  (wt %) | Ref. |
| --- | --- | --- | --- | --- | --- | --- | --- |
| Carbon Fibers | PW | 57.0 | 48.8 | 199.4 | 199.2 | 91.2 | [1] |
| Graphene | PW | 52.9 | 46.2 | 210.6 | 209.2 | 92.4 | [2] |
| Metal | PW | 63.5 | 54.5 | 83.6 | 98.8 | 95 | [3] |
| Silicon | PW | 59.2 | 49.8 | 112.9 | 122.7 | 70 | [4] |
| Adromischus Cooperi  Carbon | PW | 61.9 | 56.5 | 133.1 | 147.7 | 95.0 | [5] |
| CNT Sponge | PW | 91.0 | 27.0 | - | 138.2 | - | [6] |
| Boron Nitride | PW | 51.7 | 47.6 | 135.1 | 142.8 | 74.0 | [7] |
| MXene | PW | 60.0 | 33.7 | 131.2 | 129.5 | 80.3 | [8] |
| This work | PW | 45.6 | 34.9 | 261.7 | 259.7 | 96.2 | - |

**Table S3.** Solar-thermal conversion efficiency calculation data sheet.

| r (mW/cm^2^) | ∆𝐻𝑓 (J /g) | 𝑚 (g) | S (cm^2^) | ∆T (s) | 𝜃 (%) |
| --- | --- | --- | --- | --- | --- |
| 20 | 259.7 | 0.9548 | 4π | 1003 | 98.4 |
| 40 | 259.7 | 0.9548 | 4π | 1019 | 48.4 |
| 60 | 259.7 | 0.9548 | 4π | 910 | 36.1 |
| 80 | 259.7 | 0.9548 | 4π | 926 | 26.6 |
| 100 | 259.7 | 0.9548 | 4π | 923 | 21.4 |

**Enthalpy efficiency and relative enthalpy efficiency**

$\lambda=\frac{{\Delta H_{m}}_{(PCC)}}{{\Delta H_{m}}_{(PW)}}\times100\%$ (Eq. S1)

$\eta=\frac{{\Delta H_{m}}_{(PCC)}}{{\Delta H_{m}}_{(PW)}\times w}\times100\%$ (Eq. S2)

Where ${\Delta H_{m}}_{(PCC)}$ and ${{\Delta H}_{m}}_{(PW)}$ are respectively the melting enthalpy of MXene-K^+^@PW PCC and the melting enthalpy of pure PW, w represents the mass fraction of PW in MXene-K^+^@PW.

$\theta=\frac{m\times{\Delta H_{m}}_{(PCC)}}{r\times s\times\Delta T}$ (Eq. S3)

where m, ΔHm_(PCC)_, r, s, and ΔT are the weight, phase change crystallization enthalpy, light density, surface area, and duration of the MK3@PW phase change process, respectively.

**Electromagnetic shielding parameters**

The film samples were cut into the rectangular shape with a dimension of 22.9 × 10.2 mm^2^ for measurements. The reflection (R), transmission (T), and absorption (A) coefficients were obtained by calculating the scattering parameters (S_11_ and S_21_). The total electromagnetic interference shielding values (SE_T_) can be obtained by Eqs. 3-8

The calculation of formulas of EMI shielding parameters

SE_T_=SE_R_ + SE_A_ + SE_M_ (Eq. S4)

R + A + T =1 (Eq. S5)

R= (S_11_)^2^ = (S_22_)^2^ (Eq. S6)

T= (S_12_)^2^ = (S_21_)^2^ (Eq. S7)

SE_R_=-10 ^log (1-R)^ (Eq. S8)

SE_A_=-10 ^log (T/(1-R))^ (Eq. S9)

Where SE_R_ is the reflection value, SE_A_ is the absorption value, and SE_M_ is the multiple internal reflection value. The SE_M_ can be negligible at the time of SE_T_ ≥ 15 dB. To compare the effectiveness of shielding materials equitably, density and thickness of the materials were also taken into account. The related equations were described as:

SE/t= SE / thickness = dB/mm (Eq. S10)

The EMI shielding efficiency (%) can be obtained as Eq. S11:

Shielding efficiency (%) = 100 – 100/10^SE/10^ (Eq. S11)

**Table S4.** Comparison of EMI SE/t (dB/mm) and Energy storage density (J/g) for different PCCs.

| PCC | Crystallization enthalpy (J/g) | EMI SE  (dB) | Thickness  (mm) | SSE/t  (dB/mm) | Ref. |
| --- | --- | --- | --- | --- | --- |
| PP/CNTs/Fe_3_O_4_/PW | 130.1 | 41.2 | 2 | 20.6 | [9] |
| Epoxy/MXene/PEG | 79.1 | 64.7 | 3 | 21.6 | [10] |
|  | 96.8 | 38.3 | 1 | 38.3 |  |
| MF/PEG | 127.6 | 30.5 | 2 | 15.7 | [11] |
| Carbon scaffold/PW | 112.6 | 24.3 | 1.5 | 16.2 | [12] |
|  | 121.4 | 21.0 | 1.5 | 14 |  |
| Graphene/AgNWs/PEG | 163.3 | 71.1 | 6 | 11.9 | [13] |
| Cellulose nanocrystal/konjac glucomannan /MXene/PW | 215.7 | 45 | 10.1 | 4.5 | [14] |
| Wood/Fe3O4/PEG | 109.5 | 55 | 1 | 55 | [15] |
| MK2@PW (This work) | 250.8 | 52.3 | 0.5 | 104.6 | - |
| MK3@PW (This work) | 259.7 | 57.7 | 0.5 | 115.4 | - |

**Reference**.

1. N. Sheng, R. Zhu, K. Dong, T. Nomura, C. Zhu, Y. Aoki, H. Habazaki, T. Akiyama. Vertically aligned carbon fibers as supporting scaffolds for phase change composites with anisotropic thermal conductivity and good shape stability. J. Mater.Chem. A. **7**(9), 4934-4940 (2019). <https://doi.org/10.1039/c8ta11329g>

2. W. Ren, L. Cao, D. Zhang. Composite phase change material based on reduced graphene oxide/expanded graphite aerogel with improved thermal properties and shape‐stability. Int. J. Energ. Res. **44**(1), 242-256 (2019). <https://doi.org/10.1002/er.4900>

3. L. Zhang, G. Feng. A one-step-assembled three-dimensional network of silver/polyvinylpyrrolidone (pvp) nanowires and its application in energy storage. Nanoscale. **12**(19), 10573-10583 (2020). <https://doi.org/10.1039/d0nr00991a>

4. H. Gao, L. Bo, P. Liu, D. Chen, A. Li, Y. Ou, C. Dong, J. Wang, X. Chen, C. Hou, W. Dong, G. Wang. Ambient pressure dried flexible silica aerogel for construction of monolithic shape-stabilized phase change materials. Sol. Energ. Mat. Sol. C. **201**, 110122 (2019). <https://doi.org/10.1016/j.solmat.2019.110122>

5. C. Li, B. Xie, Z. He, J. Chen, Y. Long. 3d structure fungi-derived carbon stabilized stearic acid as a composite phase change material for thermal energy storage. Renew. Energ. **140,** 862-873 (2019). <https://doi.org/10.1016/j.renene.2019.03.121>

6. L. Chen, R. Zou, W. Xia, Z. Liu, Y. Shang, J. Zhu, Y. Wang, J. Lin, D. Xia, A. Cao. Electro- and photodriven phase change composites based on wax-infiltrated carbon nanotube sponges. ACS Nano. **6**(12), 10884-10892 (2012). <https://doi.org/10.1021/nn304310n>

7. Z. Qian, H. Shen, X. Fang, L. Fan, N. Zhao, J. Xu. Phase change materials of paraffin in h-bn porous scaffolds with enhanced thermal conductivity and form stability. Energ. Build. **158,** 1184-1188 (2018). <https://doi.org/10.1016/j.enbuild.2017.11.033>

8. X. Lu, H. Huang, X. Zhang, P. Lin, J. Huang, X. Sheng, L. Zhang, J.-p. Qu. Novel light-driven and electro-driven polyethylene glycol/two-dimensional mxene form-stable phase change material with enhanced thermal conductivity and electrical conductivity for thermal energy storage. Compos. Part B-Eng. **177,** 107372 (2019). <https://doi.org/10.1016/j.compositesb.2019.107372>

9. X. Li, M. Sheng, S. Gong, H. Wu, X. Chen, X. Lu, J. Qu. Flexible and multifunctional phase change composites featuring high-efficiency electromagnetic interference shielding and thermal management for use in electronic devices. Chem. Eng.J. **430**, 13298 (2022). <https://doi.org/10.1016/j.cej.2021.132928>

10. H. Liu, R. Fu, X. Su, B. Wu, H. Wang, Y. Xu, X. Liu. Mxene confined in shape-stabilized phase change material combining enhanced electromagnetic interference shielding and thermal management capability. Compos. Sci. Technol. **210**, 108835 (2021). <https://doi.org/10.1016/j.compscitech.2021.108835>

11. Y. J. He, Y. W. Shao, Y. Y. Xiao, J. H. Yang, X. D. Qi, Y. Wang. Multifunctional phase change composites based on elastic mxene/silver nanowire sponges for excellent thermal/solar/electric energy storage, shape memory, and adjustable electromagnetic interference shielding functions. ACS Appl. Mater. Interfaces. **14**(4), 6057-6070 (2022). <https://doi.org/10.1021/acsami.1c23303>

12. M. Zhou, J. Wang, Y. Zhao, G. Wang, W. Gu, G. Ji. Hierarchically porous wood-derived carbon scaffold embedded phase change materials for integrated thermal energy management, electromagnetic interference shielding and multifunctional application. Carbon. **183**, 515-524 (2021). <https://doi.org/10.1016/j.carbon.2021.07.051>

13. C. Wu, L. Zeng, G. Chang, Y. Zhou, K. Yan, L. Xie, B. Xue, Q. Zheng. Composite phase change materials embedded into cellulose/polyacrylamide/graphene nanosheets/silver nanowire hybrid aerogels simultaneously with effective thermal management and anisotropic electromagnetic interference shielding. Adv. Composites Hybrid, Ma. **6**(1), 31 (2023). <https://doi.org/10.1007/s42114-022-00618-9>

14. Y. Cao, Z. Zeng, D. Huang, Y. Chen, L. Zhang, X. Sheng. Multifunctional phase change composites based on biomass/mxene-derived hybrid scaffolds for excellent electromagnetic interference shielding and superior solar/electro-thermal energy storage. Nano Res. **15**(9), 8524-8535 (2022). <https://doi.org/10.1007/s12274-022-4626-6>

15. S. Liu, M. Sheng, H. Wu, X. Shi, X. Lu, J. Qu. Biological porous carbon encapsulated polyethylene glycol-based phase change composites for integrated electromagnetic interference shielding and thermal management capabilities. J. Mater. Sci. Technol. **113**, 147-157 (2022). <https://doi.org/10.1016/j.jmst.2021.11.008>
